# Upregulation of β-catenin signaling represents a single common pathway leading to the various phenotypes of spinal degeneration and pain

**DOI:** 10.1038/s41413-023-00253-0

**Published:** 2023-04-14

**Authors:** Ke Lu, Qingyun Wang, Hua Jiang, Jun Li, Zhou Yao, Yongcan Huang, Jianquan Chen, Yejia Zhang, Guozhi Xiao, Xueyu Hu, Zhuojing Luo, Liu Yang, Liping Tong, Di Chen

**Affiliations:** 1 Research Center for Computer-aided Drug Discovery, Shenzhen Institute of Advanced Technology, Chinese Academy of Sciences, Shenzhen, China; 2 Faculty of Pharmaceutical Sciences, Shenzhen Institute of Advanced Technology, Chinese Academy of Sciences, Shenzhen, China; 3 Division of Spine Surgery, the First Affiliated Hospital of Guangxi Medical University, Nanning, Guangxi China; 4 Department of Orthopedic Surgery, Perelman School of Medicine, University of Pennsylvania, Philadelphia, PA USA; 5 Institute of Orthopedics, Xijing Hospital, the Fourth Military Medical University, Xi’an, China; 6 Department of Spine Surgery, Peking University Shenzhen Hospital, Shenzhen, China; 7 Department of Clinical Medicine, School of Medicine, Zhejiang University City College, Hangzhou, Zhejiang China; 8 Departments of Physical Medicine & Rehabilitation, Perelman School of Medicine, University of Pennsylvania, Philadelphia, PA USA; 9 School of Medicine, Southern University of Science and Technology, Shenzhen, China

**Keywords:** Bone, Pathogenesis

## Abstract

Spine degeneration is an aging-related disease, but its molecular mechanisms remain unknown, although elevated β-catenin signaling has been reported to be involved in intervertebral disc degeneration. Here, we determined the role of β-catenin signaling in spinal degeneration and in the homeostasis of the functional spinal unit (FSU), which includes the intervertebral disc, vertebra and facet joint and is the smallest physiological motion unit of the spine. We showed that pain sensitivity in patients with spinal degeneration is highly correlated with β-catenin protein levels. We then generated a mouse model of spinal degeneration by transgenic expression of constitutively active β-catenin in *Col2*^+^ cells. We found that β-catenin-TCF7 activated the transcription of CCL2, a known critical factor in osteoarthritic pain. Using a lumbar spine instability model, we showed that a β-catenin inhibitor relieved low back pain. Our study indicates that β-catenin plays a critical role in maintaining spine tissue homeostasis, its abnormal upregulation leads to severe spinal degeneration, and its targeting could be an avenue to treat this condition.

## Introduction

Degenerative disc disease is an aging-related disease and is a leading cause of low back pain (LBP). It is marked by metabolic disturbances in the matrix of intervertebral discs (IVDs) and facet joints.^1^ Genetic factors, physical loading and aging are the major contributors to the disease.^2^ It has been shown that IVD degeneration is often associated with facet joint osteoarthritis (OA)^3^; however, the mechanisms for this association remain unknown. The lumbar facet joints form posterolateral articulations connecting the vertebral arch of one vertebra to the arch of the adjacent vertebra.^4^ In patients with LBP, recurrent rotational strains lead to changes to the discs and paired facet joints, causing loss of disc height, osteophyte formation, and hypertrophy of the facets.^5,6^ Cumulative evidence suggests that changes in any part of the spine tissue could lead to corresponding changes in the other parts. For example, loss of structural integrity of the IVDs has been shown to result in concomitant degenerative changes in the facet joints.^7–9^ The reverse is also true. Pathological abnormalities in facet joints accelerate the degenerative process of the IVDs.^10–12^ These findings suggest that spinal degeneration is a disease affecting multiple tissues in the spine, including the IVDs, vertebra, and facet joints. These three tissues constitute the structural basis of the functional spinal unit (FSU), the smallest physiological motion unit of the spine. The degenerative changes in these tissues significantly contribute to the development of LBP, the major symptom leading to individuals seeking medical treatment. Therefore, spinal degeneration could be controlled by a common molecular mechanism. Although spinal degeneration often affects the entire FSU, few studies have investigated this disease in an integrative way. Using traditional approaches to analyze disc degeneration, facet joint OA or discogenic pain separately provides an incomplete understanding of how the degenerative process can affect the entire FSU. Therefore, we believe that if a common molecular mechanism can be defined, it will bring about a fundamental breakthrough for the understanding and treatment strategy for degenerative spine disease.

β-Catenin is the key mediator of the canonical Wnt signaling pathway and plays an important role in skeletal development.^13^ Inhibition of β-catenin signaling leads to defects in postnatal cartilage development,^14^ and cartilage-specific activation or inhibition of β-catenin signaling showed that β-catenin regulates chondrocyte maturation, generation of ossification centers, and perichondrial bone formation during skeletal development.^15^ Abnormal inhibition or upregulation of β-catenin signaling in articular cartilage causes defects in cartilage development and osteoarthritis-like phenotypes.^16,17^ Upregulation of β-catenin signaling in IVD tissues also results in dysfunction of disc tissues.^18^ However, the role of β-catenin signaling in spinal degeneration has not been fully defined. In this study, we determined the role of β-catenin signaling in maintaining the homeostasis of the FSU and in the development of spinal degeneration. We generated transgenic mice that express constitutively active β-catenin in *Col2*-expressing cells in the spine and analyzed their spinal phenotype. We found multiple spinal degeneration-related phenotypes in these mice, including severe defects in disc tissues, high bone mass in vertebral bone adjacent to the disc, facet joint OA-like alterations and low back pain. We investigated the molecular signaling by which β-catenin activation leads to pain. Using a lumbar spine instability (LSI) model,^19^ we also demonstrated that a β-catenin inhibitor protected mice from disc degeneration and relieved pain sensation. Together, our findings provide novel insights into the mechanisms of spinal degeneration and low back pain, suggesting that elevated β-catenin signaling acts as a common molecular mechanism to induce defects in all three tissue components of the FSU and that targeting this pathway could be a potential therapeutic intervention for spinal degeneration disease.

## Results

### Upregulation of β-catenin protein levels in patients with disc degeneration and facet joint osteoarthritis

**Fig. 1:**
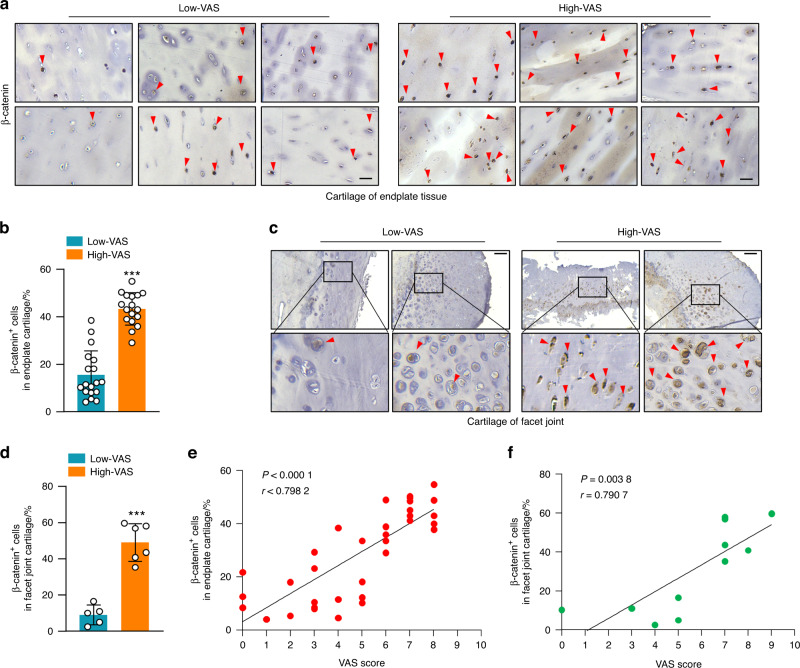
Upregulation of β-catenin protein levels in patients with disc degeneration and facet joint osteoarthritis. **a**, **b** Representative immunohistochemistry (IHC) images (**a**) showing the expression of β-catenin^+^ cells (**b**) in endplate cartilage in the high-VAS and low-VAS groups (scale bar 50 μm). (High-VAS group: *n* = 17, Low-VAS group: *n* = 18). **c**, **d** Representative IHC images (**c**) showing the expression of β-catenin^+^ cells (**d**) in facet joint cartilage in the high-VAS and low-VAS groups (scale bar, 100 μm). (High-VAS group: *n* = 6, Low-VAS group: *n* = 5). **e** Correlation analysis between the number of β-catenin^+^ cells in endplate cartilage and VAS scores in individuals with disc degeneration. (*n* = 35). **f** Correlation analysis between the number of β-catenin^+^ cells in facet joint cartilage and VAS scores in individuals with disc degeneration. (*n* = 11). *P < 0.05; **P < 0.01; ***P < 0.001. Data are represented as the mean ± SD. Two-tailed unpaired Student’s *t* test analysis (b and d) and Spearman’s correlation analysis (**e**, **f**)

To evaluate the role of β-catenin signaling in maintaining the homeostasis of the FSU and the development of spinal degeneration, we collected IVD and facet joint tissues from age-matched individuals with spinal degeneration from Xijing Hospital (Table S1). It has been reported that spinal degeneration shows a close correlation with low back pain.^20^ According to the visual analog scale (VAS), a psychometric pain response scale, 35 patients were recruited and divided into low VAS (18 cases, VAS ≤ 5) and high VAS (17 cases, VAS ≥ 6) groups. We then determined the correlation between β-catenin protein levels and pain experience in patients with spinal degeneration. We found that the patients with high β-catenin protein levels at the growth plate (GP) and endplate (EP) cartilage areas (Fig. 1a, b) and at facet joints (Fig. 1c, d) had high-VAS scores, while those with low-VAS scores had relatively lower levels of β-catenin protein levels in these tissues (Fig. 1a–d). The β-catenin protein levels at the GP and EP and the facet joint were positively correlated with high-VAS scores (Fig. 1e, f), indicating that upregulation of β-catenin levels is positively associated with severe LBP.

### Activation of β-catenin signaling leads to IVD degeneration and vertebral sclerosis

**Fig. 2:**
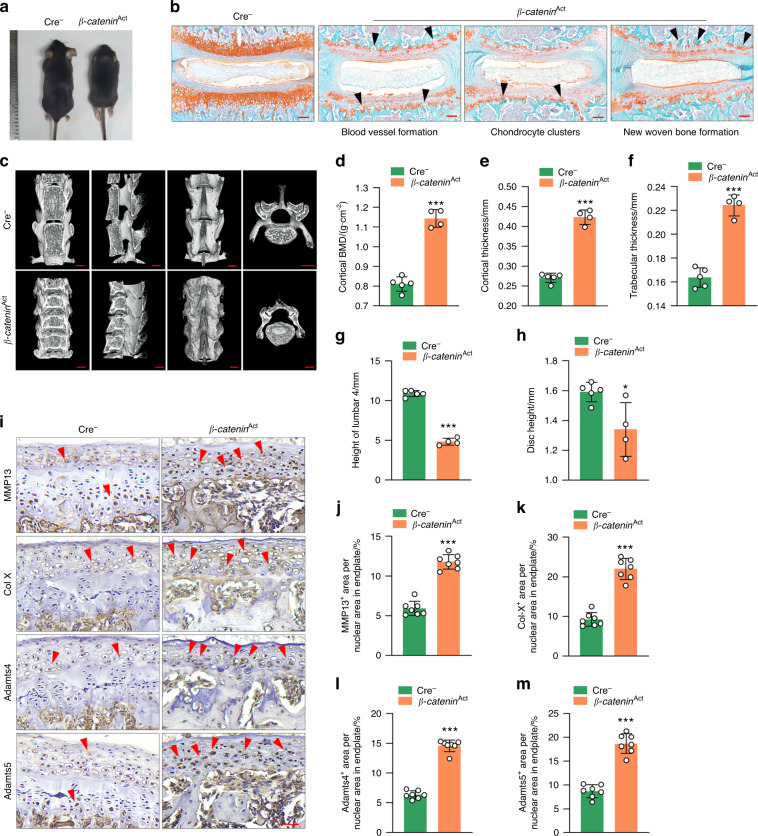
Activation of β-catenin signaling leads to intervertebral disc degeneration and vertebral sclerosis. **a** Representative image showing the size of 3-month-old *β-catenin*^Act^ mice and their Cre^−^ littermates (scale bar 100 μm). **b** Representative Safranin O/Fast Green staining of disc tissue in 5-week-old *β-catenin*^Act^ mice and their Cre^−^ littermates (scale bar 100 μm). Arrowheads indicate cell cluster formation, blood vessel invasion and new woven bone formation (*n* = 7). **c** Representative μCT images of vertebral bone of *β-catenin*^Act^ mice and their Cre^−^ littermates (scale bar 2.5 mm). **d**–**f** Histomorphometric analysis to measure cortical bone mineral density (BMD) (**d**), cortical thickness (**e**) and trabecular thickness (**f**) in 3-month-old *β-catenin*^Act^ mice and their Cre^−^ littermates (Cre^−^: *n* = 5, *β-catenin*^Act^: *n* = 4). **g**, **h** The height of the 4th lumbar vertebra (**g**) and the disc height (**h**) in 3-month-old *β-catenin*^Act^ mice and their Cre^−^ littermates (Cre^−^: *n* = 5, *β-catenin*^Act^: *n* = 4). **i**–**m** Representative IHC images (**i**) (scale bar 20 μm) of MMP13^+^ (**j**), Col-X^+^ (**k**), Adamts4^+^ (**l**) and Adamts5^+^ (**m**) areas in the endplate of 3-month-old *β-catenin*^Act^ mice and their Cre^−^ littermates. Arrowheads indicate positive cells by IHC (*n* = 7). *P < 0.05; **P < 0.01; ***P < 0.001. Data are represented as the mean ± SD. Two-tailed unpaired Student’s *t* test analysis (**d**–**h**, **j**–**m**)

To determine the role of β-catenin signaling in spinal degeneration, we generated transgenic mice, *β-catenin*(*ex3*)^Col2ER^ (also called *β-catenin*^Act^), that express a constitutively active form of β-catenin in *Col2-*expressing cells by breeding *β-catenin*(*ex3*)^flox/flox^ mice with *Col2-CreER* transgenic mice. We found that the size of 3-month-old *β-catenin*^Act^ mice was smaller than that of their Cre^−^ littermates (Fig. 2a). Our previous study data demonstrated that *Col2-CreER* transgenic mice could efficiently target GP and EP cells and inner annular fibrosus (AF) cells but not nucleus pulposus (NP) cells.^21,22^ To further analyze β-catenin activation in mice, we performed Safranin O/Fast Green staining and found loss of GP and EP cells and cell cluster formation in the GP and EP areas of *β-catenin*^Act^ mice (Fig. 2b). Blood vessel invasion and new woven bone formation were also observed at the areas where GP and EP tissues were originally located in 5-week-old *β-catenin*^Act^ mice (Fig. 2b). Micro-CT analysis showed increased vertebral bone mass, especially at the area adjacent to the IVD, and osteophyte formation, leading to narrowing of the disc space in 3-month-old *β-catenin*^Act^ mice (Fig. 2c). The length of the femur was decreased, while the femur bone volume showed an increase vs. tissue volume (BV/TV), trabecular thickness and cortical thickness in 3-month-old *β-catenin*^Act^ mice compared with those in Cre^−^ littermates (Fig. S1a–i). The results of histomorphometric analysis showed that cortical bone mineral density (BMD), cortical thickness and trabecular thickness were significantly greater in 3-month-old *β-catenin*^Act^ mice than in Cre^−^ littermates (Fig. 2d–f). The height of the 4th lumbar vertebra and the disc height were significantly lower in 3-month-old *β-catenin*^Act^ mice (Fig. 2g, h). Immunohistochemistry (IHC) results further demonstrated that the expression levels of MMP13, Col-X, Adamts4 and Adamts5 were significantly greater at the EP of *β-catenin* activation mice compared to Cre^−^ controls (Fig. 2i–m).

### Vascular invasion and nerve ingrowth at the GP and EP areas of the IVD in β-catenin-activated mice

**Fig. 3:**
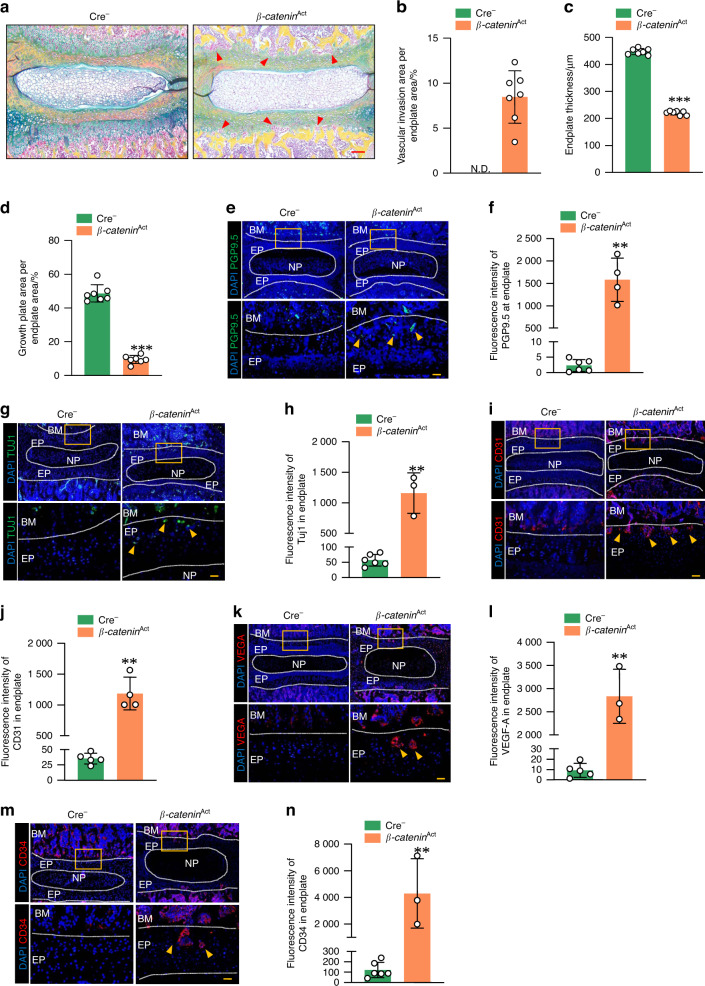
Vascular invasion and nerve ingrowth at the endplate area of the IVD in β-catenin activation transgenic mice. **a** Representative Alcian blue/hematoxylin & Orange G staining (scale bar 100 μm) to visualize blood vessel invasion and new woven bone formation in 5-week-old *β-catenin*^Act^ mice and their Cre^−^ littermates (*n* = 7). **b–d** Histological analysis to measure vascular invasion (**b**), endplate thickness (**c**) and growth plate area (**d**) in 5-week-old *β-catenin*^Act^ mice and their Cre^−^ littermates (*n* = 7). **e–n** Representative IF images (scale bar 25 μm) visualizing PGP9.5 (**e**, **f**), TUJ1 (**g**, **h**), VEGF-A (**i, j**), CD31 (**k**, **l**) and CD34 (**m**, **n**) expression at the endplate area in 5-week-old *β-catenin*^Act^ mice and their Cre^−^ littermates (Cre^−^: *n* = 6, *β-catenin*^Act^: *n* = 3 or 4). Arrowheads indicate a positive IF signal in the endplate area in *β-catenin*^Act^ mice. BM: bone marrow, EP endplate, NP nucleus pulposus. *P < 0.05; **P < 0.01; ***P < 0.001. Data are represented as the mean ± SD. Two-tailed unpaired Student’s *t* test analysis (**b**–**d**, **f**, **h**, **j**, **l**, **n**)

To determine the role of β-catenin signaling in vascular invasion and nerve ingrowth at the endplate, we performed Alcian blue/Hematoxylin & Orange G staining in samples from *β-catenin*^Act^ mice and found loss of growth plate cartilage cells and endplate cells and vascular invasion in *β-catenin*^Act^ mice compared with the Cre^−^ littermates (Fig. 3a–d). The results of IF staining and quantification analyses showed that high expression of neuronal markers (PGP9.5 and TUJ1) was detected in the EP area of 5-week-old *β-catenin*^Act^ mice (Fig. 3e–h). In addition, high expression of markers of vascular formation (VEGF-A, CD31 and CD34) was also detected at the endplate area in 5-week-old *β-catenin*^Act^ mice (Fig. 3i–n). These findings suggest that nerve ingrowth and vascular invasion are induced by β-catenin activation in the IVD.

### Severe facet joint OA in β-catenin activation mice

**Fig. 4:**
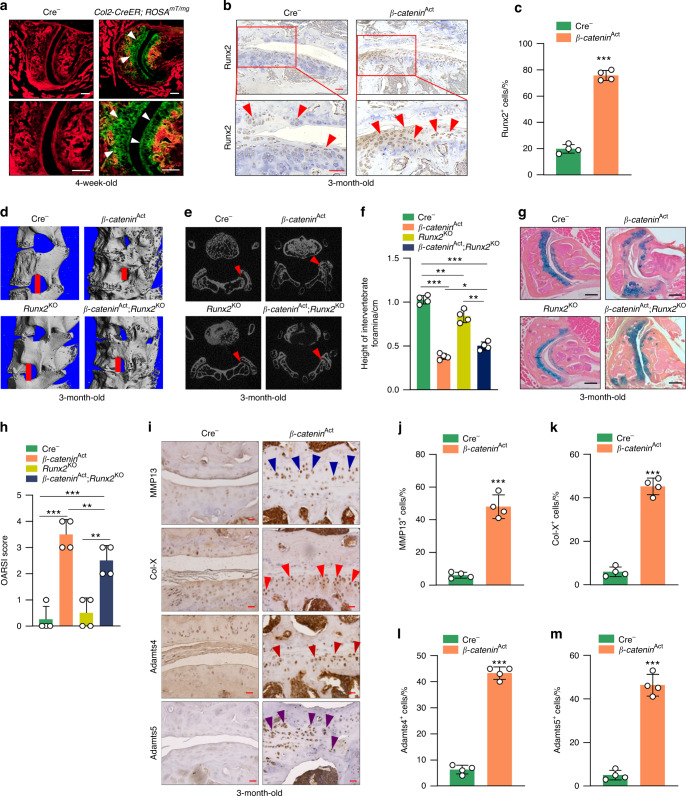
Severe facet joint osteoarthritis in β-catenin activation transgenic mice. **a** Representative IF images showing *Col2-CreER* targeting in articular cartilage of the facet joint (*n* = 4) (scale bar 50 μm). Arrowheads indicate articular cartilage of the facet joint. **b**, **c** Representative IHC images showing expression of Runx2^+^ in the facet joint in 3-month-old *β-catenin*^Act^ mice and their Cre^−^ littermates (*n* = 4) (scale bar 15 μm). **d–f** Representative μCT images showing osteophyte formation (**d**), joint space narrowing (**e**) and the height of intervertebral foramina (**f**) in 3-month-old *β-catenin*^Act^ mice, *Runx2*^*KO*^ mice, *β-catenin*^*Act*^*; Runx2*^*KO*^ mice and their Cre^−^ littermates (*n* = 4). **g**, **h** Representative Alcian blue/hematoxylin staining (scale bar 30 μm) showing histomorphometric changes (**g**) and OARSI scores (**h**) in the facet joint in 3-month-old *β-catenin*^Act^ mice, *Runx2*^*KO*^ mice, *β-catenin*^*Act*^*; Runx2*^*KO*^ mice and their Cre^−^ littermates (*n* = 4). **i–m** Representative IHC images (**i**) (scale bar 15 μm) showing the expression of MMP13^+^ (**j**), Col-X^+^ (**k**), Adamts4^+^ (**l**) and Adamts5^+^ (**m**) cells in the facet joint in 3-month-old *β-catenin*^Act^ mice and their Cre^−^ littermates (*n* = 4). *P < 0.05; **P < 0.01; ***P < 0.001. Data are represented as the mean ± SD. Two-tailed unpaired Student’s *t* test analysis (**b**, **j**–**m**). One-way ANOVA followed by Tukey’s posttest for multiple comparisons (**f**, **h**)

To fully characterize changes in spinal degeneration, we determined the facet joint OA phenotype in β-catenin-activated mice. By IF analysis of *Col2-CreER; ROSA*^*mT/mG*^ mice, we found that *Col2-CreER* mice could efficiently target the articular cartilage of the facet joint (Fig. 4a). Runx2 is a key signaling molecule in OA development^23,24^; thus, we then determined Runx2 expression by IHC and found that activation of β-catenin signaling resulted in greater Runx2 expression in the facet joint of the β-catenin activation mice compared to their Cre^−^ littermates (Fig. 4b, c). Foraminal stenosis is one of the main reasons for LBP,^25^ so we next evaluated the morphology of the intervertebral foramina—the opening between every two vertebrae where the nerve roots exit the spine—in 3-month-old *β-catenin*^Act^ mice by μCT analysis. We found osteophyte formation, joint space narrowing and reduced height of the intervertebral foramina in *β-catenin*^Act^ mice compared to their Cre^−^ littermates, while *β-catenin*^Act^; *Runx2*^KO^ mice showed partial reversal of these phenotypes (Fig. 4d–f). We then performed Alcian blue/hematoxylin staining of the facet joint in *β-catenin*^Act^ mice and found severe defects in the articular cartilage of the facet joint, including loss of cartilage matrix and articular chondrocytes, disorganized articular cartilage tissue and increased OARSI (Osteoarthritis Research Society International) scores, a semiquantitative measure of OA, and osteophyte formation in *β-catenin* activation mice compared to their Cre^−^ littermates. Deletion of *Runx2* in *β-catenin*^Act^; *Runx2*^KO^ double mutant mice partially reversed the increase in OARSI scores seen in the *β-catenin*^Act^ mice (Fig. 4g, h). These results were consistent with our previous study in knee joint OA.^17^ To clarify the role of β-catenin activation in cartilage matrix destruction in the facet joint, we performed an IHC assay and found that the expression levels of MMP13, Col-X, Adamts4 and Adamts5 were significantly greater in *β-catenin* activation mice than in their Cre littermates (Fig. 4i–m).

### Activation of β-catenin causes low back pain

**Fig. 5:**
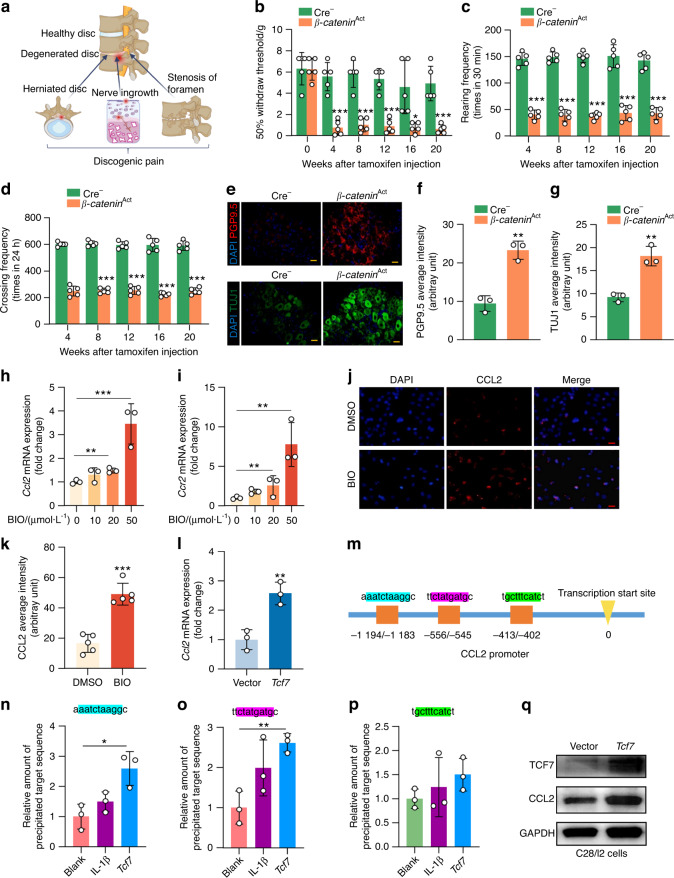
Activation of β-catenin causes discogenic pain. **a** A schematic diagram illustrating three reasons for discogenic pain. **b**–**d** The von Frey test and LABORAS test showing the sensitivity to mechanical allodynia (**b**), rearing number (**c**) and crossing frequency (**d**) in *β-catenin*^Act^ mice compared with their Cre^−^ littermates with tamoxifen injection at the indicated time points (*n* = 5). **e–g** Representative IF images (**e**) (scale bar 25 μm) showing PGP9.5 (**f**) and TUJ1 (**g**) expression in dorsal root ganglia (DRG) tissues in *β-catenin*^Act^ mice and Cre^−^ littermates (*n* = 3). **h**, **i** qRT‒PCR of CCL2 (**h**) and CCR2 (**i**) mRNA levels in C28/I2 cells treated with the GSK-3β inhibitor BIO (0, 10, 20, 50 μmol·L^−1^) (*n* = 3), with the level in BIO (0 μmol·L^−1^) arbitrarily set to 1. **j**, **k** Representative IF images (**j**) (scale bar 25 μm) and quantification (**k**) of CCL2 average intensity in C28/I2 cells treated with BIO (50 μmol·L^−1^) (*n* = 5). **l** qRT‒PCR of *CCL2* mRNA levels in C28/I2 cells transfected with TCF7 plasmid (*n* = 3), with the level in the vector group arbitrarily set to 1. **m–p** Chromatin immunoprecipitation (ChIP) assays for binding of TCF7 to the CCL2 promoter. The binding regions were predicted by JASPAR—A database of transcription factor binding profiles (https://jaspar.genereg.net/) (**m**). ChIP assays were performed using HEK293T cells treated with IL-1β or transfected with *TCF7* plasmid. ChIP-enriched DNA was subjected to qPCR (**n**–**p**), with the level in the blank group arbitrarily set to 1. **q** Representative Western blots of TCF7 and CCL2 protein expression in C28/I2 cells transfected with TCF7 plasmid (*n* = 3). GAPDH expression was detected as a loading control. *P < 0.05; **P < 0.01; ***P < 0.001. Data are represented as the mean ± SD. Two-tailed unpaired Student’s t test analysis (**f**, **g**, **k**, **l**). One-way ANOVA followed by Tukey’s posttest for multiple comparisons (**b**–**d**, **h**, **i**, **n**–**p**)

Herniation of the disc tissue, stenosis of the foramen and excessive endplate innervation are potential causes of LBP (Fig. 5a). We thus performed a series of tests to determine whether activation of β-catenin could increase pain sensation. Using the von Frey test, we found that sensitivity to mechanical allodynia was greater in β-catenin-activated mice than in Cre^−^ controls (Fig. 5b). The rearing number (Fig. 5c) and crossing frequency (Fig. 5d) were significantly lower in the β-catenin activation mice than in the Cre^−^ controls, indicating that spontaneous activity was lower in the β-catenin activation mice.

To further examine LBP induced by activation of β-catenin, we isolated bilateral L3–5 dorsal root ganglia (DRG) and performed IF and qPCR to detect changes in the expression of neuronal markers. We found that the protein expression of PGP9.5 and TUJ1 and the mRNA expression of *TrpA1*, *Ngf*, *Calca*, *Scn9a*, *TNF-α* and *IL-1β* were all greater in β-catenin-activated mice than in Cre^−^ controls (Figs. 5e–g, S2a–f). Furthermore, as CCL2-CCR2 signaling has been reported to play a key role in regulating pain in OA,^26^ we performed IF analysis and quantification and found that the expression of CCL2 was much greater at the endplate area in 5-week-old *β-catenin*^Act^ mice than in Cre^−^ controls (Fig. S3a, b), suggesting that chondrocyte-derived CCL2 could stimulate excess innervation at the endplate area, contributing to LBP.

To determine the mechanism by which β-catenin regulates CCL2 expression in chondrocytes, we used the GSK-3β inhibitor BIO to increase β-catenin levels in C28/I2 cells—a human chondrocyte cell line—as GSK-3β acts to destabilize β-catenin, leading to its protein degradation. We found that the expression levels of both *Ccl2* and *Ccr2* mRNA were increased in a dose-dependent manner in chondrocytes after cells were treated with BIO (Fig. 5h, i). By qPCR assay, we found an upregulation of β-catenin downstream target genes (*Axin2* and *Dkk1*) and other chemokine genes (*Ccl3, Ccr1, Ccr5, Cxcr1, Cxcr2* but not *Ccl5*) in a dose-dependent manner in C28/I2 cells treated with BIO (Fig. S4a–h). We also found by Western blotting that BIO upregulated β-catenin and CCL2 protein expression in C28/I2 cells (Fig. S5a–c). Additionally, IF results showed that BIO upregulated CCL2 expression in C28/I2 cells (Fig. 5j, k).

Once in the nucleus, β-catenin binds to TCF/LEF proteins to activate the transcription of β-catenin target genes.^27^ We thus transfected C28/I2 cells with *Tcf7* expression plasmids and found that forced expression of *Tcf7* resulted in upregulated expression of *Axin2*, *Dkk1*, *Ccl2*, *Ccl3*, *Ccr1*, *Ccr2*, *Ccr5*, *Cxcr1*, and *Cxcr2*, but not *Ccl5*, in C28/I2 cells (Figs. 5l, S6a–j). IF results showed that transfection of *Tcf7* increased CCL2 expression in C28/I2 cells (Fig. S7a, b).

We identified putative TCF7 binding sites in the CCL2 promoter through bioinformatics analysis (Fig. 5m). We then performed a chromatin immunoprecipitation (ChIP) assay using HEK293T cells treated with IL-1β or transfected with the *Tcf7* plasmid. ChIP-enriched DNA was subjected to qPCR analysis. We found that TCF7 binds to the −1 194/−1 183 and −556/−545 regions of the *CCL2* promoter (Fig. 5n–p). These results were also confirmed by Western blot and IF analyses. The results of Western blot analysis demonstrated that transfection of *Tcf7* in C28/I2 cells significantly increased CCL2 expression in C28/I2 cells (Figs. 5q, S8a, b).

### β-catenin inhibition reverses LBP in an LSI mouse model

**Fig. 6:**
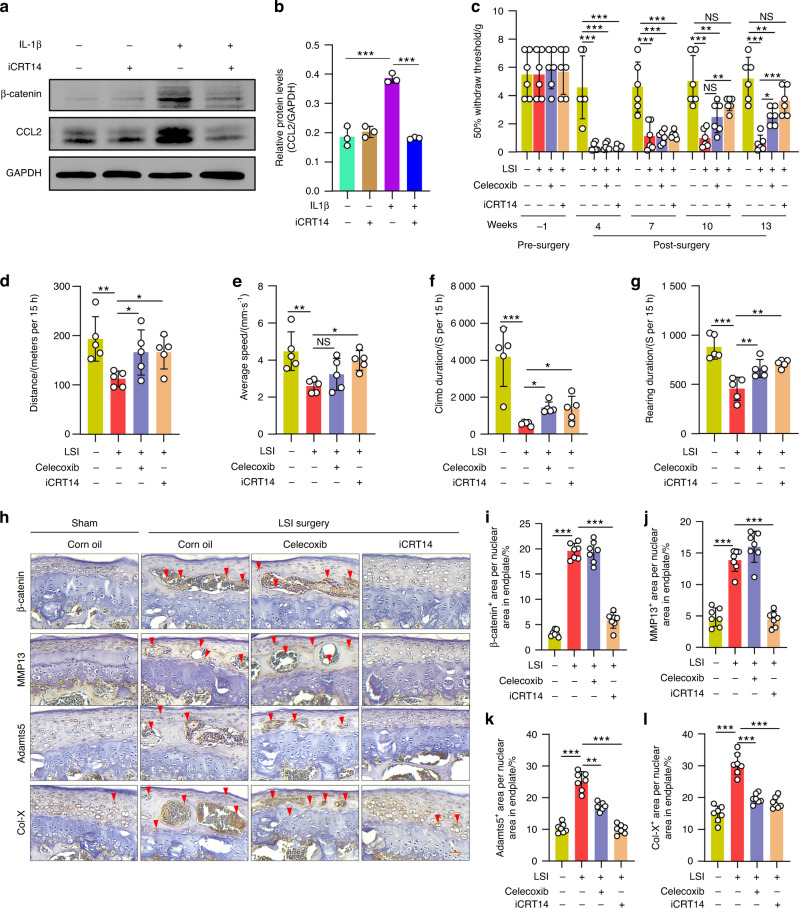
β-catenin inhibition reverses discogenic pain caused by LSI in mice. **a**, **b** Representative Western blots (**a**) and quantification of CCL2 (**b**) protein expression in C28/I2 cells treated with IL-1β (20 ng·mL^−1^) or iCRT14 (50 μmol·L^−1^), as indicated (*n* = 3). GAPDH expression was detected as a loading control. **c–g** The von Frey test and LABORAS test showing the sensitivity to mechanical allodynia (**c**), distance (**d**), average speed (**e**), climb duration (**f**) and rearing duration (**g**) in the LSI mice treated with iCRT14 (50 mg·kg^−1^) or celecoxib (10 mg·kg^−1^) (positive control) (*n* = 5). **h–l** Representative IHC images (**h**) (scale bar 50 μm) showing the expression of β-catenin^+^ (**i**), MMP13^+^ (**j**), Adamts5^+^ (**k**) and Col-X^+^ (**l**) areas in the endplate of lumber spine instability (LSI) mice treated with iCRT14 (50 mg·kg^−1^) or celecoxib (10 mg·kg^−1^) (positive control). Corn oil was treated as a negative control. Arrowheads indicate positive cells in IHC (*n* = 7). *P < 0.05; **P < 0.01; ***P < 0.001. Data are represented as the mean ± SD. One-way ANOVA followed by Tukey’s posttest for multiple comparisons (**b**–**g**, **i**–**l**)

As a β-catenin/TCF inhibitor, iCRT14 can interfere with TCF binding to DNA.^28^ To determine if iCRT14 could alleviate LBP, we first treated C28/I2 cells with iCRT14 (50 μmol·L^−1^) and found that iCRT14 did not affect the expression of *Tcf7*, *Axin2*, *Dkk1*, *Ccl2*, *Ccl3*, *Ccl5*, *Ccr1*, *Ccr2*, *Ccr5*, *Cxcr1* and *Cxcr2* in C28/I2 cells in a noninflammatory environment (i.e., when the cells were not treated with IL-1β) (Fig. S9a–k). In contrast, we found that iCRT14 inhibited IL-1β-induced gene expression, including *Axin2*, *Dkk1*, *Ccl2*, *Ccl3*, *Ccl5*, *Ccr1*, *Ccr2*, *Ccr5*, *Cxcr1* and *Cxcr2*, after C28/I2 cells were treated with IL-1β (Fig. S10a–j). In addition, we also found that treatment with IL-1β increased CCL2 protein expression in C28/I2 cells, and this effect was blocked by iCRT14 (Fig. 6a, b).

We then performed LSI surgery in 12-week-old C57 mice to mimic a spinal degeneration phenotype.^19^ Four weeks after surgery, LSI mice were treated with iCRT14 (50 mg·kg^−1^) or Celebrex (10 mg·kg^−1^) (an anti-inflammatory factor that acts as a positive control) for 6 weeks. We found that LSI caused LBP (as determined by a reduction in von Frey scores). Treatment with iCRT14 (6 weeks, 50 mg·kg^−1^ per injection for a total of 12 injections) or treatment with Celebrex (6 weeks, 10 mg·kg^−1^ per injection for a total of 12 injections) significantly reversed LSI-induced mechanical allodynia at 10 and 13 weeks after surgery (Fig. 6c). Analyses of changes in spontaneous activities, including distance traveled, average speed, climb duration and rearing duration, showed decreases in the LSI mice at baseline. However, treatment with iCRT14 reversed the decreases in spontaneous activities caused by LSI surgery (Fig. 6d–g). We also recorded the motion track of the mice. LSI caused decreases in spontaneous activities, including locomotion duration, locomotion frequency, climb frequency, rear frequency, and max speed, and increases in immobility frequency and immobility duration. Treatment with iCRT14 significantly reversed these reduced spontaneous activities to a normal status 13 weeks after LSI surgery (Fig. S11a–h). The results of IHC analysis showed that the expression of β-catenin, MMP13, Col-X and Adamts5 was significantly decreased at the endplate in the LSI mice treated with iCRT14 (Fig. 6h–l). We also found that the expression of CD31 and PGP9.5, by IF assay, was increased at the endplate of the LSI mice, and was decreased by treatment with iCRT14 (Fig. S12a–d).

## Discussion

In this study, we found that abnormal upregulation of β-catenin signaling in the spine leads to a degenerative phenotype in multiple tissues of the FSU, including IVD degeneration, vertebral sclerosis, facet joint OA, and low back pain. We investigated the molecular mechanisms involved in the degenerative processes and found that Runx2, MMP13 and CCL2 are key downstream mediators of β-catenin in *Col2*-expressing cells in the spine. In facet joint tissue, Runx2 was upregulated in β-catenin activation mice, suggesting that Runx2 is a downstream target gene of β-catenin signaling. Deletion of *Runx2* under a β-catenin activation background can only partially reverse the facet joint OA phenotype, suggesting that β-catenin may be able to use other transcription factor(s) to mediate its signaling to activate genes encoding matrix degradation enzymes. Our findings indicate that β-catenin plays a critical role in spinal degeneration and that activation of β-catenin signaling under pathological conditions leads to severe defects in multiple tissues within the FSU.

The IVD is a complex structure that provides flexibility to the vertebral column and sustains the weight applied through the spine. The vertebrae are covered superiorly and inferiorly by the GP and EP cartilage, which allow for the intake of nutrients and the removal of waste products of the NP. The IVD is formed by the peripheral AF. The inner region of the AF is characterized by a transition from type I to type II collagen and increased proteoglycan content^29^ and circumferentially encloses the NP, which is a highly hydrated structure that is predominantly composed of aggrecan. It is not known whether degeneration of the IVD is initiated from defects in the NP tissue or from tissues outside the NP, such as GP and EP cartilage or the AF. However, as *Col2-CreER* mice, used in the current study, only target GP and EP cartilage and AF tissues, but not NP tissue,^21^ our findings suggest that IVD degeneration could be initiated from tissues outside the NP. Changes in NP tissue are secondary to alterations in GP and EP cartilage or AF tissues.

LBP has been recognized as the main symptom of spinal degeneration^20,30,31^; however, the source of pain and how nerve signaling is activated by local pain mediators have not been defined. In this study, we found that striking nerve ingrowth occurs after the loss of GP and EP cartilage tissues in β-catenin activation mice, indicating that nerve ingrowth at the region of the GP and EP cartilage could be a potential source for LBP. In addition, we also found that the size of the intervertebral foramina is reduced due to large amounts of osteophyte formation in surrounding tissues, which could repress the nerve tissue inside the intervertebral foramina, resulting in pain sensation. It is well known that β-catenin plays an important role in neuropathic pain.^32–35^ However, the role of β-catenin in LBP and related molecular mechanisms have not been determined. In the current study, we found that β-catenin interacts with TCF7 and directly regulates CCL2 gene transcription. CCL2 was shown to mediate OA-related pain in previous studies.^26,36^ In the current study, we demonstrated that CCL2 is a direct downstream target gene of β-catenin signaling, suggesting that CCL2 mediates LBP during the development of spinal degeneration.

In a magnetic resonance imaging (MRI) study evaluating the relationship between facet joint OA and IVD degeneration, the former was rarely found in the absence of the latter but tended to be most pronounced at spinal levels associated with advanced IVD degeneration.^37^ Similar findings were also reported by other studies showing a positive correlation between IVD degeneration and facet joint OA.^8,10,13^ However, clear evidence regarding changes in the entire FSU during spinal degeneration has not been thoroughly investigated. Consistent with these reports, in the current study, we clearly demonstrated that activation of β-catenin signaling in *Col2*-expressing cells in the spine leads to degenerative changes in all tissues of the FSU as well as increased pain sensation.

There are several limitations in the current study. 1) Although we clearly demonstrated that abnormal activation of β-catenin signaling leads to degenerative changes in all tissues in FSU, we do not know if this is always true for spinal degeneration caused by alterations in other signaling molecules. Further follow-up studies are needed. 2) Although we found a positive correlation between pain sensation and high β-catenin protein levels in disc and facet joint tissues in patients with spinal degeneration, this study needs to be further validated using large numbers of patient samples. Current enrolled patient numbers are relatively small, especially the facet joint samples.

In summary, our findings indicate that there is a common signaling pathway controlling the degenerative process, which may represent an effective drug target for the treatment of spine degeneration and its associated pain.

## Materials and methods

### Experimental design

The objective of this study was to determine the role of β-catenin signaling in maintaining spine tissue homeostasis and in the development of spine degeneration.

We first analyzed β-catenin protein levels by IHC and LBP by analyzing VAS scores in patients with spine degeneration. Correlations between β-catenin expression and VAS scores were assessed using Pearson’s rank correlation test.

We then generated β-catenin conditional activation mice (*β-catenin*^Act^) by breeding *β-catenin*(*ex3*)^flox/flox^ mice with *Col2-CreER* transgenic mice. We analyzed the spine degeneration phenotype in 3-month-old β-catenin activation mice using micro-CT, histology, IHC and IF analyses and pain-related behavior tests. Changes in spontaneous activities of β-catenin activation mice were analyzed by distance moved, speed, locomotion, immobility, climbing and rearing using the Laboratory Animal Behavior Observation Registration and Analysis System (LABORAS, Metris, Netherlands). Mechanical allodynia was determined by the von Frey test. After behavior tests, all the mice were sacrificed, and lumbar 3–5 and dorsal root ganglion (DRG) tissues were collected. Cortical BMD, cortical thickness, trabecular thickness, and the height of lumbar 4 were quantified using micro-CT reconstructed 3D images. The expression of MMP13, Col-X, Adamts4 and Adamts5 was examined by IHC. The expression of PGP9.5 and TUJ1 was analyzed by IF in DRG tissues. The mRNA levels of *TrpA1*, *Ngf*, *Calca*, *Scn9a*, *TNF-α* and *IL-1β* were detected by qPCR in DRG tissues.

Five-week-old *β-catenin*^Act^ mice and Cre littermate controls were used to determine the IVD degeneration phenotype. Activation of β-catenin signaling leads to defects in IVD, including loss of GP and EP cartilage cells, nerve ingrowth, vascular invasion and new woven bone formation at the places where GP/EP cells used to be located. Morphological analysis of disc tissue spine degeneration and vascular invasion was performed by Safranin O/Fast Green and Alcian blue/Hematoxylin & Orange G (AB/H&OG) staining in *β-catenin*^Act^ and Cre^−^ mice. The expression of nerve cell markers PGP9.5 and TUJ1 and vascular markers CD31, CD34 and VEGF-A in GR/EP areas was detected and quantified.

Twelve-week-old C57BL/6 (C57) mice underwent LSI surgery to mimic the process of spine degeneration. Sham surgery was used as a control. After surgery, all C57 mice were allowed to rest for 4 weeks following 6 weeks of iCRT14 (β-catenin inhibitor) treatment. Corn oil was used as a negative control, and celecoxib was used as a positive control to alleviate discogenic pain. After treatment, all mice were examined by mechanical allodynia (von Frey test) and spontaneous activity tests. IHC assays, including β-catenin, MMP13, Col-X, and Adamts5, were performed to evaluate changes in expression in IVD tissues.

The 293 cell line (HEK293) and C28/I2 cell line were used for in vitro molecular mechanism studies. The chemical compound BIO and TCF7 expression plasmid were used to activate β-catenin signaling in these cell lines. The β-catenin inhibitor iCRT14 was used to block β-catenin signaling in these cell lines. IL-1β was used to promote the expression of inflammation-related genes. To confirm that β-catenin signaling was successfully activated or inhibited, the downstream target genes of β-catenin signaling (*Axin2* and *Dkk1*) were detected by qPCR. CCL2 and CCR2 play a key role in pain signaling in OA.^26^ The expression of a series of chemokines, *Ccl2*, *Ccl3*, *Ccl5*, *Ccr1*, *Ccr2*, *Ccr5*, *Cxcr1* and *Cxcr2*, was also detected by qPCR in C28/I2 cells after treatment with BIO or transfection of *Tcf7*. Western blotting and IF (CCL2) were performed to examine β-catenin/TCF7-induced CCL2 transcription in chondrocytes. ChIP assays were performed to determine TCF7 binding to the CCL2 promoter in 293 cells treated with IL-1β or transfected with the *Tcf7* plasmid.

The patient recruitment, the group of mice, the outcome assessment, and the data analysis were performed in a blinded fashion.

### Human samples

The human samples and data were collected from Xijing Hospital, Xian, China. Individuals with spine degeneration who were willing to participate in the study were included according to the following inclusion and exclusion criteria:

#### Inclusion criteria

1. Patient age between 50 and 70 years old (50≤ age ≤70);
2. The patient was diagnosed with IVD degeneration based on imaging evidence;
3. The patient had symptoms of LBP;
4. The patient intended to undergo IVD surgery.

#### Exclusion criteria

1. The patient had autoimmune disease, diabetes, thyroid disease, a nervous system disorder and/or other diseases that may cause abnormal pain;
2. The patient had used hormone drugs in the past three months;
3. The patient currently had symptoms of infection;
4. The patient had previously undergone major spinal surgery.

The basic information for the participants is presented in Table S1. Before surgery, the individual’s degree of pain was evaluated by a visual analog scale (VAS). The patient’s spine tissues were then collected for pathological analysis during the surgery. The study was conducted with the approval of the Ethics Committee of Xijing Hospital (ky20222166). Informed written consent was provided by all participants.

### Animal studies

The animal protocol of this study was approved by the Ethics Committee of the Shenzhen Institute of Advanced Technology, Chinese Academy of Sciences, and all experimental methods and procedures were carried out in accordance with the approved guidelines to comply with all relevant ethical regulations for animal testing and research (IACUC No.: SIAT-IACUC-210201-YYS-LK-A1538). *β-catenin*^Act^ (*β-catenin*(*ex3*)^Col2ER^) mice were generated from *Col2a1-CreER* transgenic mice (C57BL/6 strain), as previously described,^18^ and *β-catenin*(*ex3*)^flox/flox^ mice (129/Sv strain) were generated as originally reported by Harada et al.^38^ and used in our previous studies.^17,18^ Tamoxifen (Sigma, St. Louis, MO) was administered to 2-week-old mice by i.p. injection (1 mg per 10 g body weight, for 5 days). *Runx2*^flox/flox^ mice (C57BL/6 strain) were generated as previously described.^39^ Wild-type (C57BL/6) mice were purchased from GemPharmatech (Nanjing, China). Rosa26 reporter mice (129/Sv strain) were obtained from Jackson Laboratories (Bar Harbor, ME, USA).^40^ Genotyping was performed by PCR analysis of DNA isolated from mouse tails, and Cre^−^ Littermates were used for all experiments.

The following primers were used for genotyping: Cre-specific primers: upper primer, 5′-ATCCGAAAAGAAAACGTTGA-3′; lower primer, 5′-ATCCAGGTTACGGATATAGT-3′; β-catenin (ex3). Flox-specific primers: upper primer, 5′-AGGGTACCTGAAGCTCAGCG-3′; lower primer, 5′-CAGTGGCTGACAGCAGCTTT-3′.

In this study, male and female mice were kept in a specific pathogen-free facility (SPF) in individually ventilated cages, 5 mice per cage, and the animals were on a 12 h day/night cycle and housed at a controlled temperature of 22 °C. Caging, food, and water bottles were changed weekly. Five-week-old *β-catenin*^Act^ mice were used for the detection of vascular invasion and nerve ingrowth. Twelve-week-old C57BL/6 mice were imported for LSI surgery. No criteria or exclusions were used in this study.

### The LSI model of spine degeneration in mice

Twelve-week-old C57BL/6 mice were randomly divided into the sham group, the LSI group, the LSI + celecoxib group and the LSI + iCRT14 group (*n* = 7 per group). The randomization sequence was generated by computer. Surgeries were performed in a random order, with mice being taken from alterative cages to avoid potential confounders, such as the order of treatments and measurements and animal/cage locations. After being anesthetized with ketamine and xylazine, mice were operated on to perform resection of the 3rd to 5th lumbar (L3–5) spine processes along with the supraspinous and interspinous ligaments to induce instability of the lumbar spine, as previously described.^19^ Sham operations were performed by detachment of only the posterior paravertebral muscles from the L3–5 vertebrae. Operative fields were closed with 4-0 nylon sutures.

### Behavioral assessments

The assessment of long-term movements of mice was conducted with the Laboratory Animal Behavior Observation Registration and Analysis System (LABORAS) (Metris, Netherlands) according to protocols, as previously described.^41,42^ Briefly, mice were placed in 4 individual cages with unlimited access to food and water. Assays ran for 15 h, starting at 18:00 h on the first day and ending at 9:00 h the next morning. The parameters analyzed included distance moved, speed, locomotion, immobility, climbing and rearing. Recorded data were taken and calculated using LABORAS 2.6 software (Metris, Netherlands).

To determine changes in mechanical allodynia, von Frey tests were performed using a calibrated set of von Frey filaments (North Coast Medical Inc., CA, USA), as previously described.^41^ Before a von Frey hind paw test, the mice were allowed to acclimate on a wire mesh grid for 15 min. If the mice exhibited any nocifensive behavior, including brisk paw withdrawal, licking, or shaking of the paw, either during the application of the stimulus or immediately after filament removal, it was considered a positive response. A set of von Frey filaments was used to poke from below the hind paw to calculate the 50% force withdrawal threshold using an iterative approach. The tests were performed in a blinded manner such that the investigator was not aware of the identification of the animals, as well as the study groups.

### Micro-CT analysis

We used a Scanco µCT35 scanner (Scanco Medical, Brüttisellen, Switzerland) with a 55 kVp source and 200 μAmp current for formalin-fixed mouse spines with a resolution of 6 μm. The scanned images from each group were evaluated at the same thresholds to allow 3-dimensional structural rendering of each sample. Regions of interest (ROI) of L3–5 lumbar spines were reconstructed, and their bone volumes were quantitatively assessed. Cortical BMD, cortical thickness, trabecular thickness (Tb.Th.), the height of the 4^th^ lumbar vertebra and disc height were analyzed by CTAn V1.15.4 software (Skyscan, Bruker, Belgium). For long bone analysis, we used a NEMO Micro-CT scanner (Pingsheng Healthcare Shanghai Inc., Shanghai, China) with a 90 kV source and 70 μA current for formalin-fixed mouse legs with a resolution of 10 μm. The ROI of the distal femur was reconstructed, and its bone parameters were quantitatively analyzed.

### Histology, IHC and IF

For histological staining, slides of mouse disc and facet joint (bilateral L3–5) coronal sections cut 5 μm thick were stained with Safranin O/Fast Green and Alcian blue/Hematoxylin & Orange G (AB/H&OG) for morphologic analysis. The vascular invasion area, EP thickness and growth plate area were analyzed using the ImageJ program.

For IHC staining, rehydrated coronal sections of discs and facet joints were heated at 95 °C in Antigen Unmasking Solution for 10 min and then sequentially treated with Endogenous Peroxidase Blocking Buffer (Beyotime, China), 0.5% Triton X-100, and an Avidin/Biotin Blocking Kit. After blocking with 10% normal goat serum for 1 h, sections were treated with primary antibodies, including anti-MMP13, anti-Col-X, anti-Adamts4, anti-Adamts5, anti-Runx2 and anti-β-catenin antibodies (see antibody list in Table S2), overnight at 4 °C and incubated with secondary biotinylated goat anti-rabbit or anti-mouse antibody for 30 min, followed by treatment with the VECTASTAIN Elite ABC Kit. IHC signals were revealed by ImmPACT DAB Peroxidase Substrate and analyzed using the ImageJ program.

For IF staining, rehydrated coronal sections of discs, facet joints and DRGs (bilateral L3–5) were incubated with specific primary antibodies, including those against PGP9.5, Tuj1, CD31, VEGF-A, CD34 and CCL2 (see antibody list in Table S2), and then incubated with a peroxidase-conjugated anti-fluorescein antibody. VECTASHIELD mounting medium with DAPI (Vector Laboratories, Burlingame, CA, USA) was used to mount the slides. Images of histology, IHC and IF were captured using CellSens Imaging Software (Olympus) on an Olympus BX43 microscope. The average intensity was analyzed using the ImageJ program.

### Cell culture and transfection

The human chondrocyte cell Line C28/I2 (BLUEFBIO, China) and the human embryonic kidney 293 cell line (HEK293) (ATCC, USA) were cultured according to the vendor’s instructions. C28/I2 cells were cultured in DMEM/F-12 medium (Gibco, USA), and HEK293 cells were cultured in DMEM (Gibco, USA). All media were supplemented with 10% fetal bovine serum at 37 °C under 5% CO_2_. IL-1β (20 ng·mL^−1^, P00019, Solarbio, China) was used to induce the expression of inflammation-related genes in C28/I2 cells for 12 h. BIO (1 μmol·L^−1^, S7198, Selleck, USA) was used to treat C28/I2 cells for 12 h to activate β-catenin signaling and induce β-catenin target genes. 50 μmol·L^−1^ iCRT14 (S8704, Selleck, USA) was used to treat C28/I2 cells for 12 h to inhibit β-catenin signaling. The TCF7 plasmid (40620, Addgene, USA) was transfected into C28/I2 cells or 293 cells using Lipofectamine 3000 to overexpress TCF7. qPCR, Western blotting or ChIP assays were performed 48 h after transfection. The cell lines were tested for mycoplasma-free status before they were used.

### Western blotting

After cell culture and treatment, cells were homogenized and incubated in RIPA lysis and extraction buffer supplemented with 1 mmol·L^−1^ PMSF, 1 mmol·L^−1^ Na3VO4 and protease inhibitor cocktail (Roche, Mannheim, Germany). After separation, samples were electrotransferred onto PVDF membranes (Bio-Rad, Hercules, California, USA) probed with the antibodies listed in Table S2, exposed to SuperSignal West Pico Chemiluminescent Substrate (Thermo Scientific, Waltham, Massachusetts, USA) and visualized by radiography.

### Chromatin immunoprecipitation (ChIP)

The ChIP assay was performed as previously described.^18^ Briefly, 293 cells were treated with IL-1β or transfected with TCF7 plasmid for 24 h. After harvesting and centrifugation, the cell pellet was cross-linked and isolated. The cross-linked cells were then lysed and digested by MNase for further immunoprecipitation (IP). A TCF7 antibody (sc-271453, Santa Cruz Biotechnology, USA) was used for pull-down of proteins or DNA binding with TCF7. After the DNA recovery step, the enriched DNA was subjected to qPCR. The binding region of TCF7 to the CCL2 promoter was predicted by JASPAR, a database of transcription factor binding profiles (https://jaspar.genereg.net/).

### Quantitative real-time PCR

Total RNA was extracted from cells or DRG tissues using one-phase RNA purification kits (MACHEREY-NAGEL, Duren, Germany). DNAse I-treated total RNA was reverse transcribed using an RT reagent kit (Takara Bio, Tokyo, Japan). The cDNA was amplified by PCR in a total volume of 20 μL reaction solution containing 10 pmol·L^−1^ primers (primer names and sequences are listed in Table S3).

### Drug administration

After surgery, all mice were allowed to rest for 4 weeks and then treated with the β-catenin inhibitor iCRT14 for 6 weeks. iCRT-14 (S8704) was purchased from Selleck (TX, USA) and was first dissolved in DMSO and then further dissolved in corn oil (50 mg·kg^−1^ body weight) and administered every 3 days for 6 weeks by i.p. injection. Injection of corn oil (S6701, Selleck, USA) was used as a negative control, and celecoxib (S1261, Selleck, USA) was used as a positive control. Celecoxib was also first dissolved in DMSO and then dissolved in corn oil (10 mg·kg^−1^ body weight) and delivered by i.p. injection on the same schedule as iCRT-14.

### Statistical analysis

The sample size for each experiment was determined based on our previous study^8^ and power analysis. For the power analysis, we used the formula *n* = 2(Zalpha + Zbeta)^2^/d^2^ to calculate sample numbers, where *d* = (u1 − u2)/sigma (sigma = SD). We assumed a probability of type I error, or alpha of 0.05, and a probability of type II error, or beta of 0.20, and thus obtained a value of Zalpha = 1.645, and Zbeta = 0.842, so *n* = 2(1.645 + 0.842)^2^/d^2^. Statistical analyses were conducted using Prism GraphPad Prim 8.0 software. All the data are expressed as the mean ± S.D., as indicated in the figure legends. Unpaired Student’s *t* test (for two groups), one-way ANOVA (for multiple groups with one variable factor) and two-way ANOVA (for multiple groups with two variable factors) were used followed by the Tukey‒Kramer test. Correlations were assessed using Pearson’s rank correlation test. All experiments were performed and analyzed using 3 individual samples unless otherwise mentioned. *P* < 0.05 was considered statistically significant and is denoted in the figures. The experiments were randomized, and the investigators were blinded to allocation during experiments and outcome assessment.

## Supplementary information

SUPPLEMENTAL MATERIAL

## Data Availability

The data that support the findings of this study are available in the main text or the Supplementary Materials or from the corresponding author upon reasonable request.
