# Supplementary material for: Upregulation of β-catenin signaling represents a single common pathway leading to the various phenotypes of spinal degeneration and pain
Source: Bone Res. 2023 Apr 14;11:18. doi: 10.1038/s41413-023-00253-0 (PMC10104801; doi:10.1038/s41413-023-00253-0)
Supplement: Supplementary file 1 — SUPPLEMENTAL MATERIAL [file 41413_2023_253_MOESM1_ESM.docx]

Supplementary Materials for

**Upregulation of β-catenin signaling represents a single common pathway leading to the various phenotypes of spinal degeneration and pain**

Ke Lu^1,2^, Qingyun Wang^1,2^, Hua Jiang^3^, Jun Li^4^, Zhou Yao^5^, Yongcan Huang^6^, Jianquan Chen^7^, Yejia Zhang^4,8^, Guozhi Xiao^9^, Xueyu Hu^5^, Zhuojing Luo^5^, Liu Yang^5^, Liping Tong^1^, and Di Chen^1,2^*

^1^ Research Center for Computer-aided Drug Discovery, Shenzhen Institute of Advanced Technology, Chinese Academy of Sciences, Shenzhen 518055, China;

^2^ Faculty of Pharmaceutical Sciences, Shenzhen Institute of Advanced Technology, Chinese Academy of Sciences, Shenzhen 518055, China;

^3^ Division of Spine Surgery, the First Affiliated Hospital of Guangxi Medical University, Nanning, Guangxi 530021, China;

^4^ Department of Orthopaedic Surgery, Perelman School of Medicine, University of Pennsylvania, Philadelphia, PA 19104, USA；

^5^ Institute of Orthopaedics, Xijing Hospital, the Fourth Military Medical University, Xi'an 710032, China;

^6^ Department of Spine Surgery, Peking University Shenzhen Hospital, Shenzhen 518035, China;

^7^ Department of Clinical Medicine, School of Medicine, Zhejiang University City College, Hangzhou, Zhejiang 310015, China;

^8^ Departments of Physical Medicine & Rehabilitation, Perelman School of Medicine, University of Pennsylvania; Philadelphia, PA 19104, USA；

^9^ School of Medicine, Southern University of Science and Technology, Shenzhen 518055, China.

*Corresponding author

Di Chen, email: [di.chen@siat.ac.cn]

**This PDF file includes:**

Fig. S1-12

Table S1-3


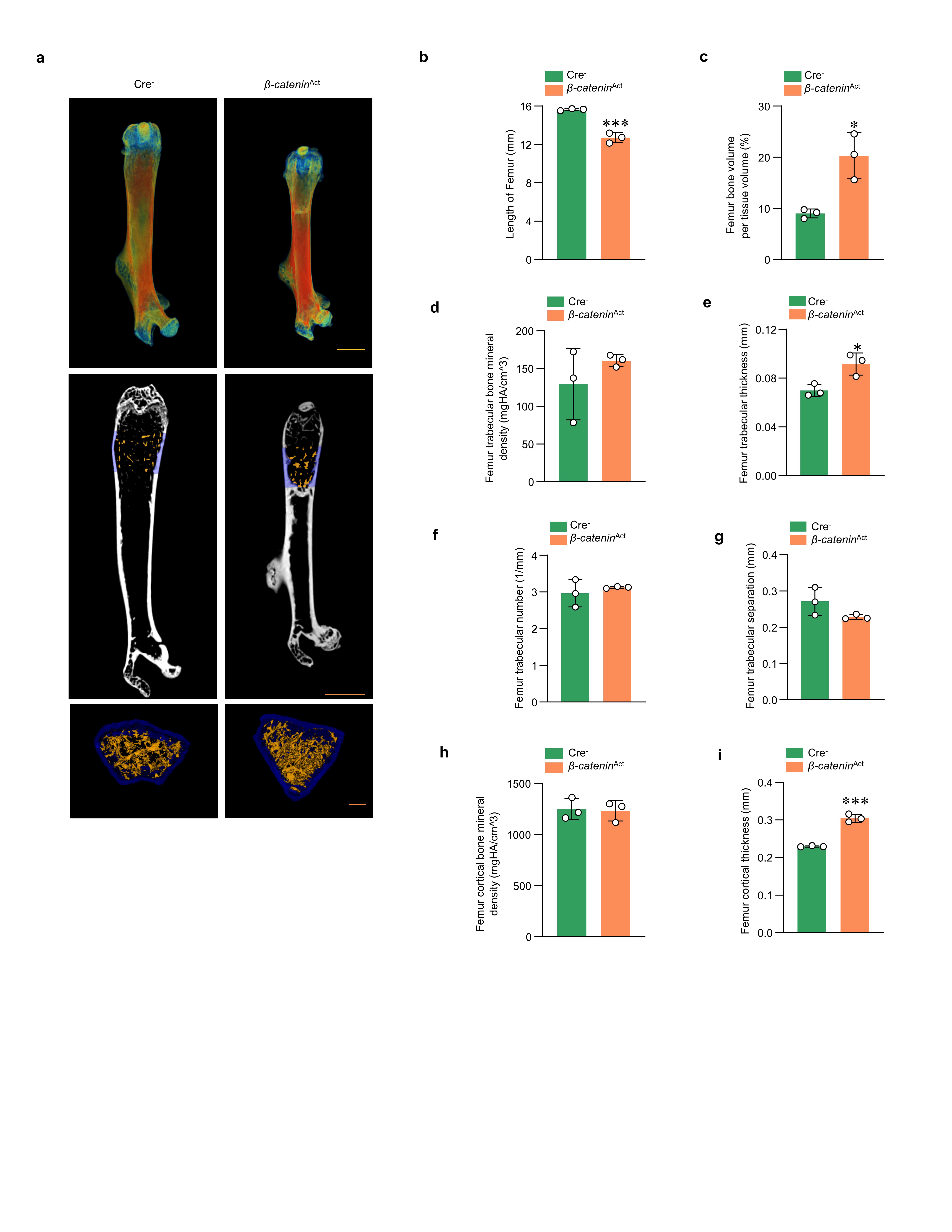


**Fig. S1** Activation of β-catenin increases femur bone volume. **a** Representative μCT images of femur of *β-catenin*^Act^ mice and their Cre^-^ littermates (scale bar 2 mm). **b-i** Histomorphometric analysis to measure length of femur (**b**), bone volume per tissue volume (**c**) trabecular bone mineral density (**d**), trabecular thickness (**e**), trabecular number (**f**), trabecular separation (**g**), cortical bone mineral density (**h**) and cortical thickness (**i**) in 3-month-old *β-catenin*^Act^ mice and their Cre^-^ littermates (Cre^-^: n = 3 and *β-catenin*^Act^: n = 3). *: *P*<0.05, **: *P*<0.01, ***: *P*<0.001. Data are represented as mean ± SD. Two-tailed unpaired Student’s *t*-test analysis (b-i).


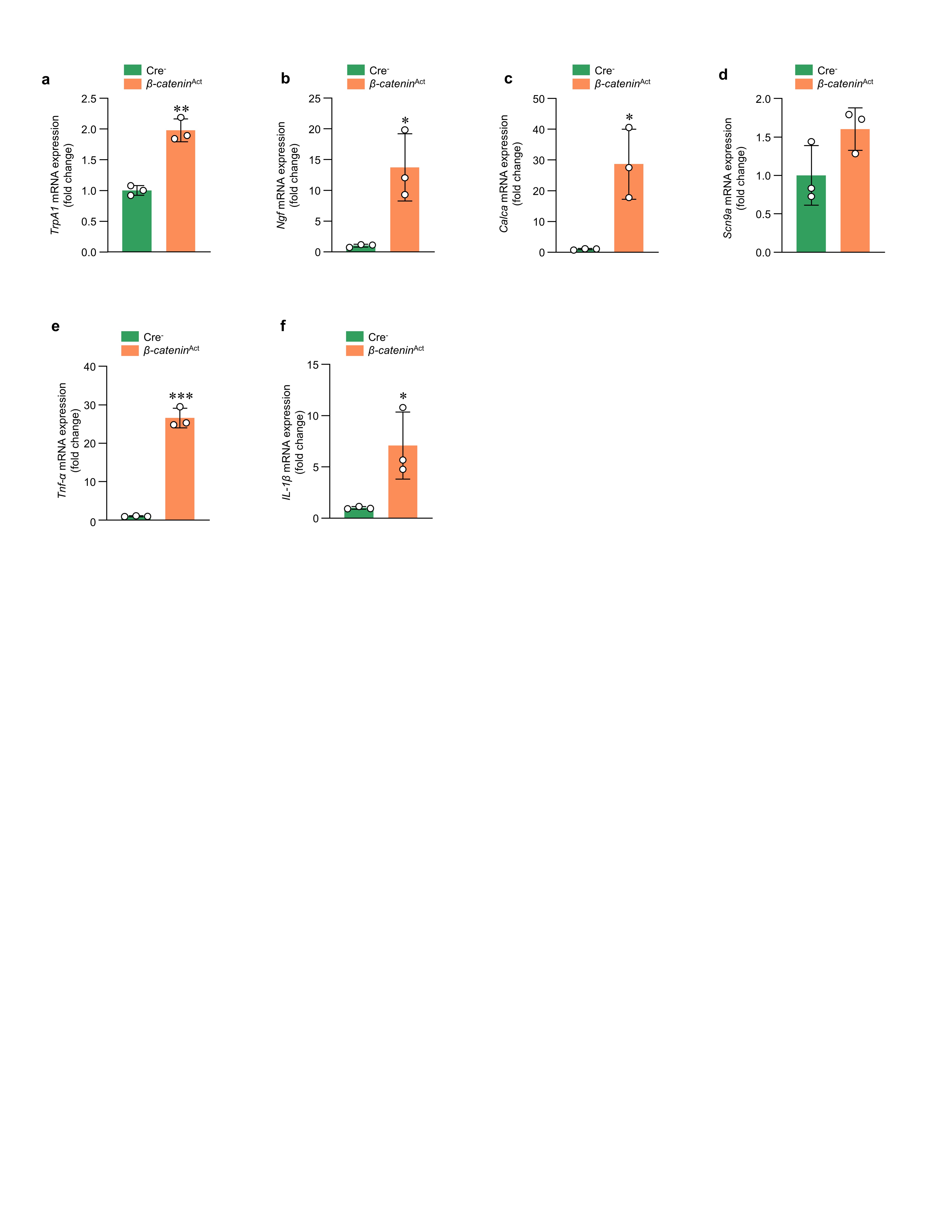


**Fig. S2** Activation of β-catenin causes low back pain (LBP). **a**-**f** mRNA expression of *TrpA1* (**a**), *Ngf* (**b**), *Calca* (**c**), *Scn9a* (**d**), *Tnf-α* (**e**) and *IL-1β* (**f**) in DRG tissues in *β-catenin*^Act^ mice and Cre^-^ littermates (n = 3). The mRNA level in Cre^-^ group was arbitrarily set to 1. *: *P*<0.05, **: *P*<0.01, ***: *P*<0.001. Data are presented as mean ± SD. Statistical analyses were conducted by two-tailed unpaired Student’s *t*-test (a-f).


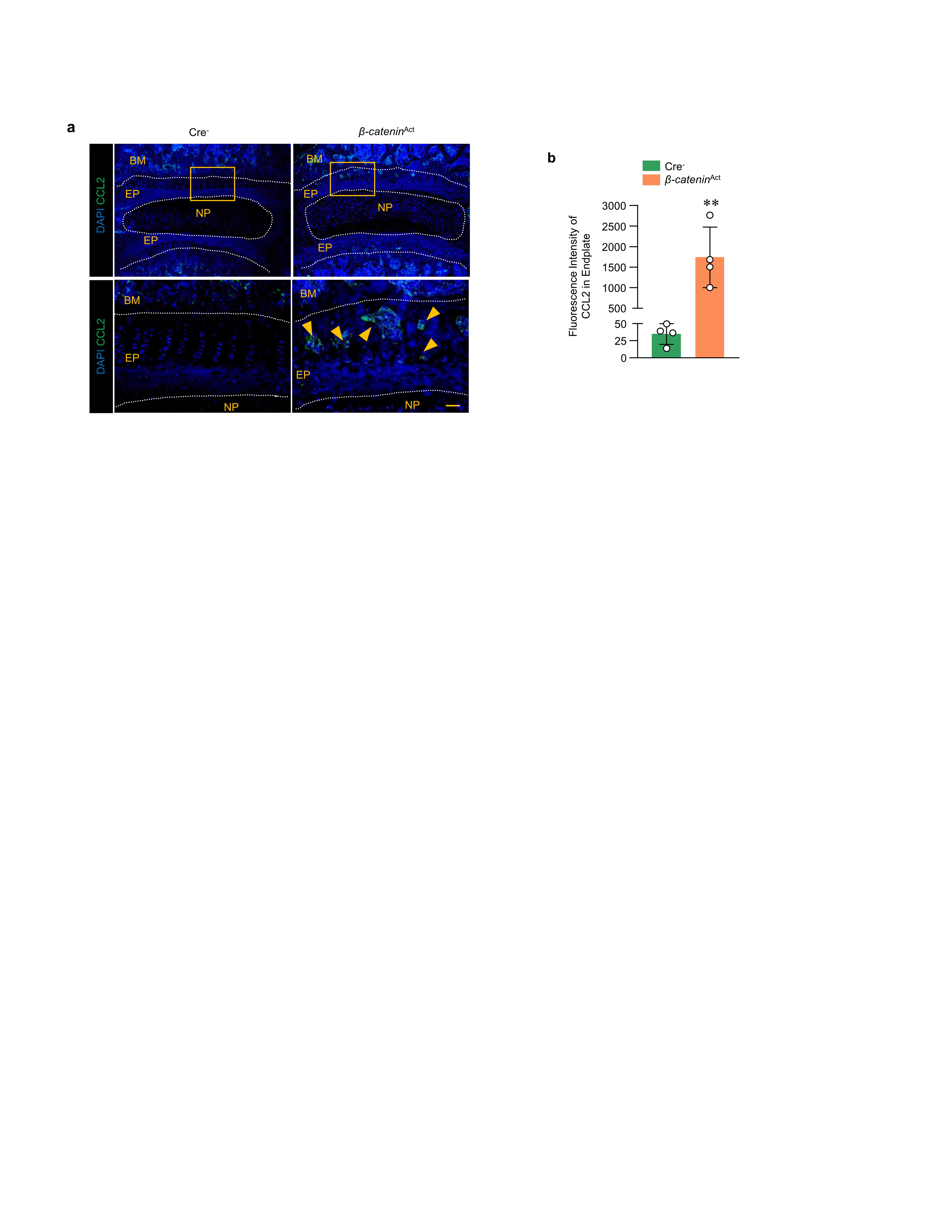


**Fig. S3** Activation of β-catenin increases CCL2 expression in GP/EP area. **a, b** Representative IF images (**a**) (scale bar 25 μm) showing CCL2 expression (**b**) at GP/EP areas in 5-week-old *β-catenin*^Act^ mice and Cre^-^ littermates (n = 4). Arrowheads indicate positive IF signal in GP/EP areas in *β-catenin*^Act^ mice. GP: growth plate, BM: bone marrow, EP: endplate, NP: nucleus pulposus. *: *P*<0.05, **: *P*<0.01, ***: *P*<0.001. Data are presented as mean ± SD. Statistical analyses were conducted by two-tailed unpaired Student’s *t*-test (b).


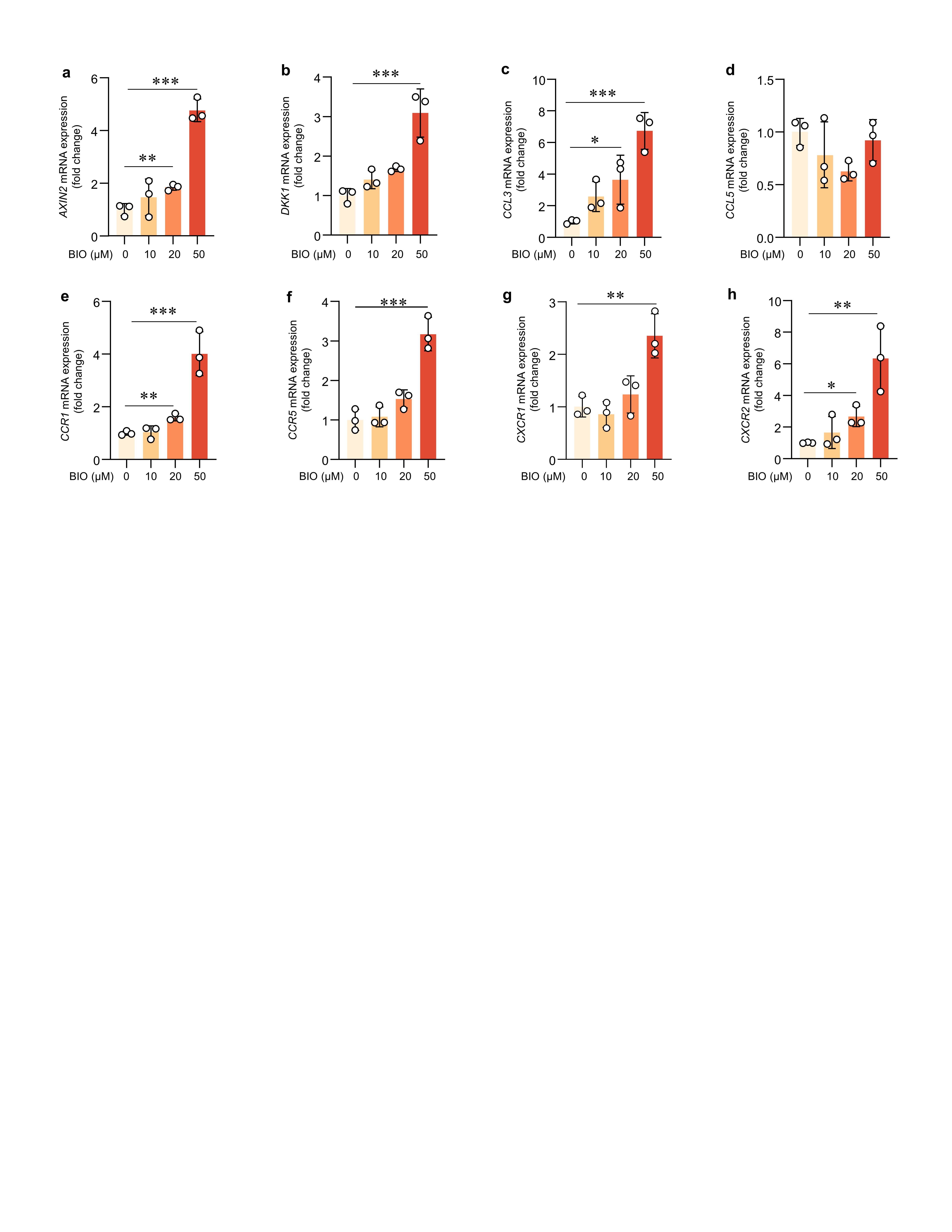


**Fig. S4** Upregulation of chemokines induced by BIO. **a-h** mRNA expression of *Axin2* (**a**), *Dkk1* (**b**), *Ccl3* (**c**), *Ccl5* (**d**), *Ccr1* (**e**), *Ccr5* (**f**), *Cxcr1* (**g**) and *Cxcr2* (**h**) determined by qPCR in C28/I2 cells treated with BIO (0, 10, 20, 50 μM) (n = 3). The mRNA level in BIO (0 μM) group was arbitrarily set to 1. *: *P*<0.05, **: *P*<0.01, ***: *P*<0.001. Data are presented as mean ± SD. Statistical analyses were conducted by one-way ANOVA followed by Tukey’s post-test for multiple comparisons (a-h).


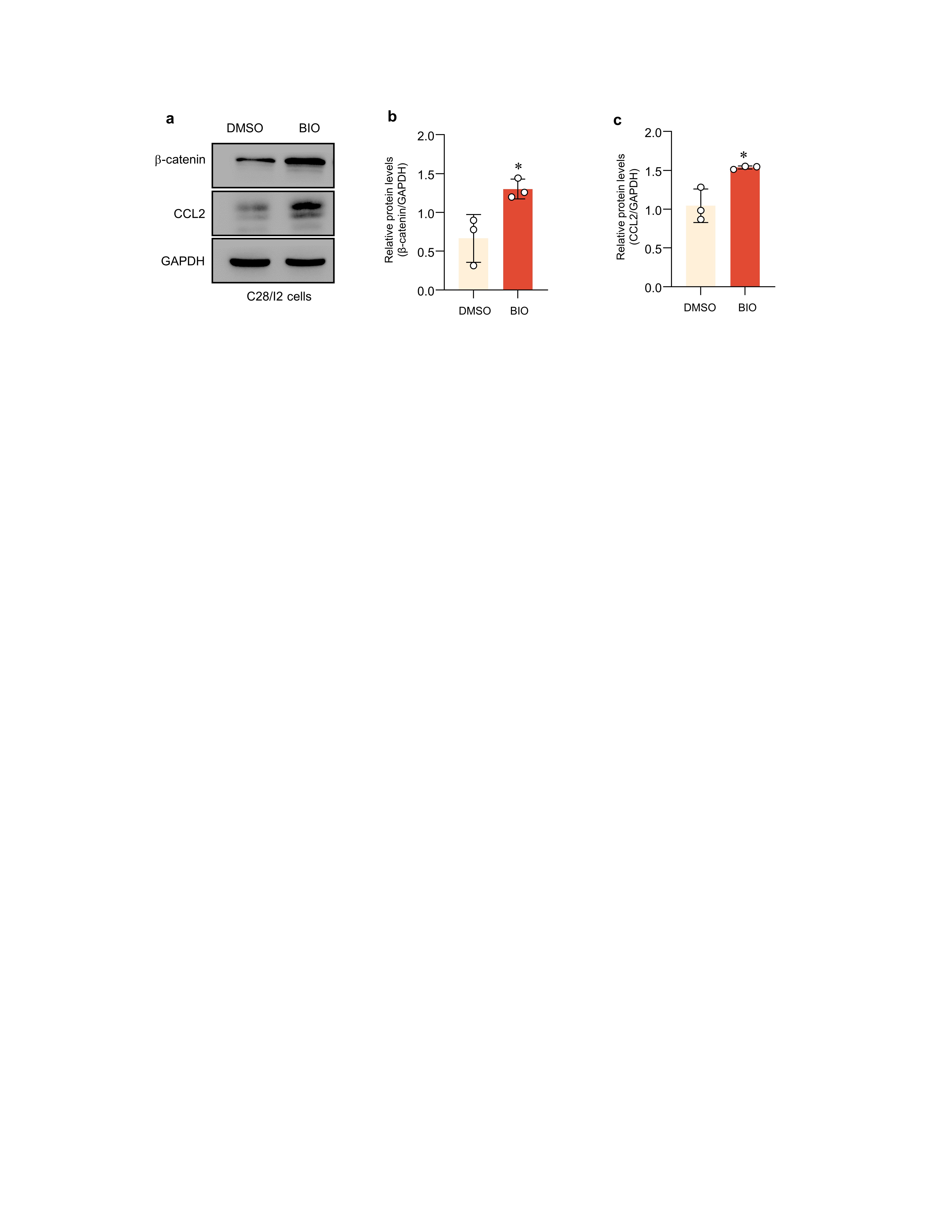


**Fig. S5** Upregulation of CCL2 induced by BIO. **a-c** Representative Western blots (**a**) and quantification of β-catenin (**b**) and CCL2 (**c**) protein levels in C28/I2 cells treated with BIO (50 μM) (n = 3). GAPDH expression was detected as a loading control. *: *P*<0.05, **: *P*<0.01, ***: *P*<0.001. Data are presented as mean ± SD. Statistical analyses were conducted by two-tailed unpaired Student’s *t*-test (b and c).


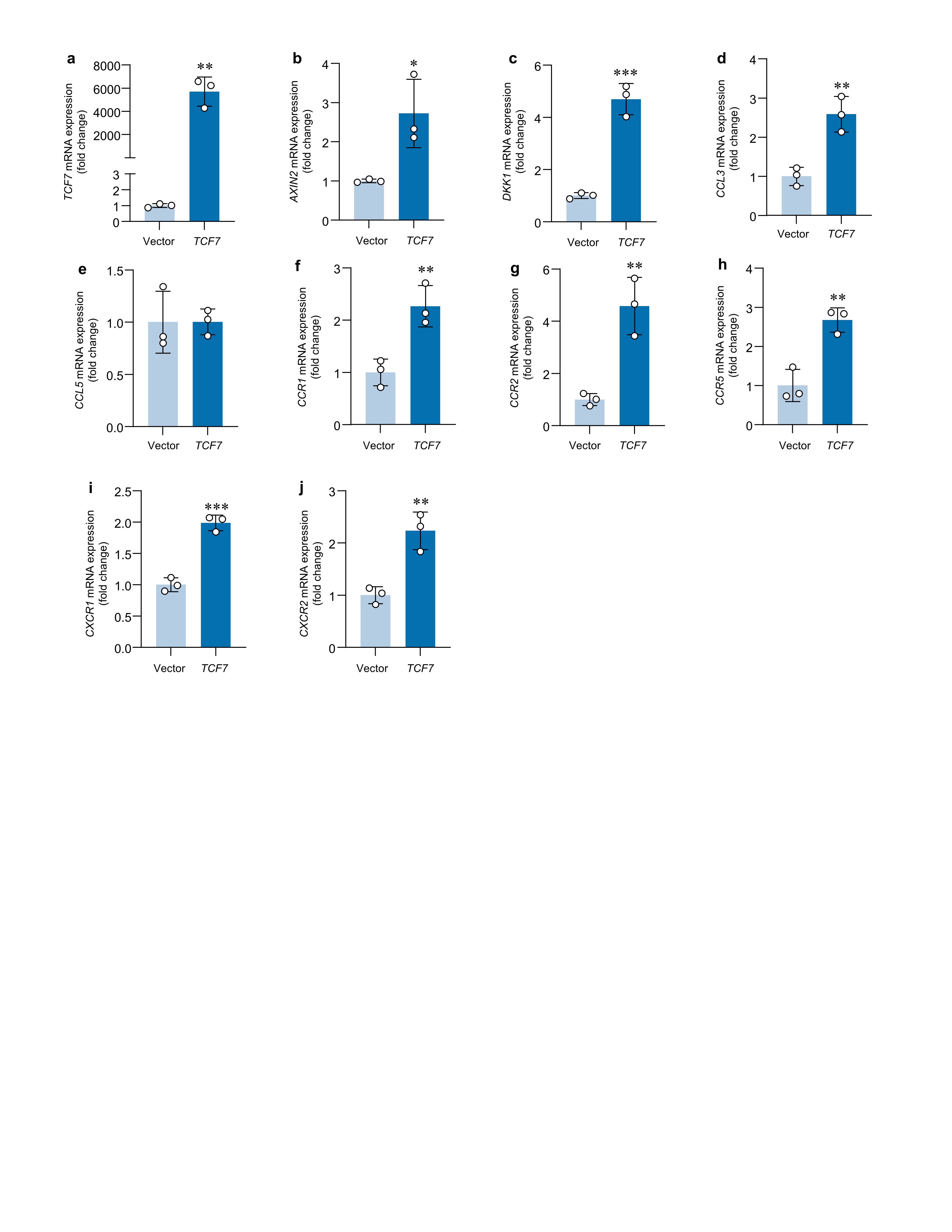


**Fig. S6** Upregulation of chemokines induced by *Tcf7* plasmid transfection. **a-j** mRNA expression of *Tcf7* (**a**), *Axin2* (**b**), *Dkk1* (**c**), *Ccl3* (**d**), *Ccl5* (**e**), *Ccr1* (**f**), *Ccr2* (**g**), *Ccr5* (**h**), *Cxcr1* (**i**) and *Cxcr2* (**j**) by qPCR analysis in C28/I2 cells transfected with *Tcf7* plasmid (n = 3). The mRNA level in vector control group was arbitrarily set to 1. *: *P*<0.05, **: *P*<0.01, ***: *P*<0.001. Data are presented as mean ± SD. Statistical analysis was conducted by two-tailed unpaired Student’s *t*-test (a-j).


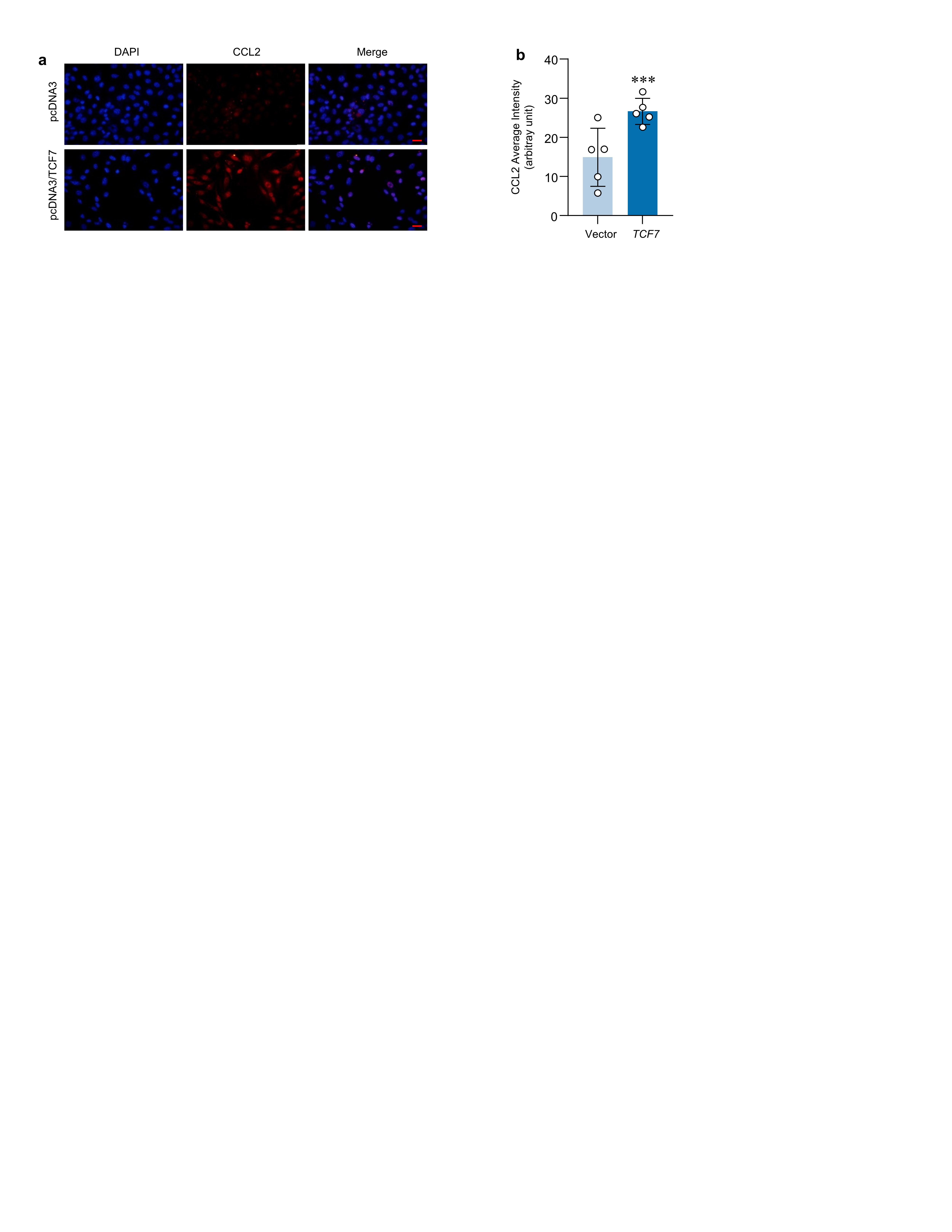


**Fig. S7** Upregulation of CCL2 expression in C28/I2 cells transfected with *Tcf7* expression plasmid. **a, b** Representative IF images (**a**) (scale bar 25 μm) and quantification (**b**) of CCL2 average intensity in C28/I2 cells transfected with *Tcf7* expression plasmid (50 μM) (n = 5). *: *P*<0.05, **: *P*<0.01, ***: *P*<0.001. Data are presented as mean ± SD. Statistical analysis was conducted using two-tailed unpaired Student’s *t*-test (b).

**
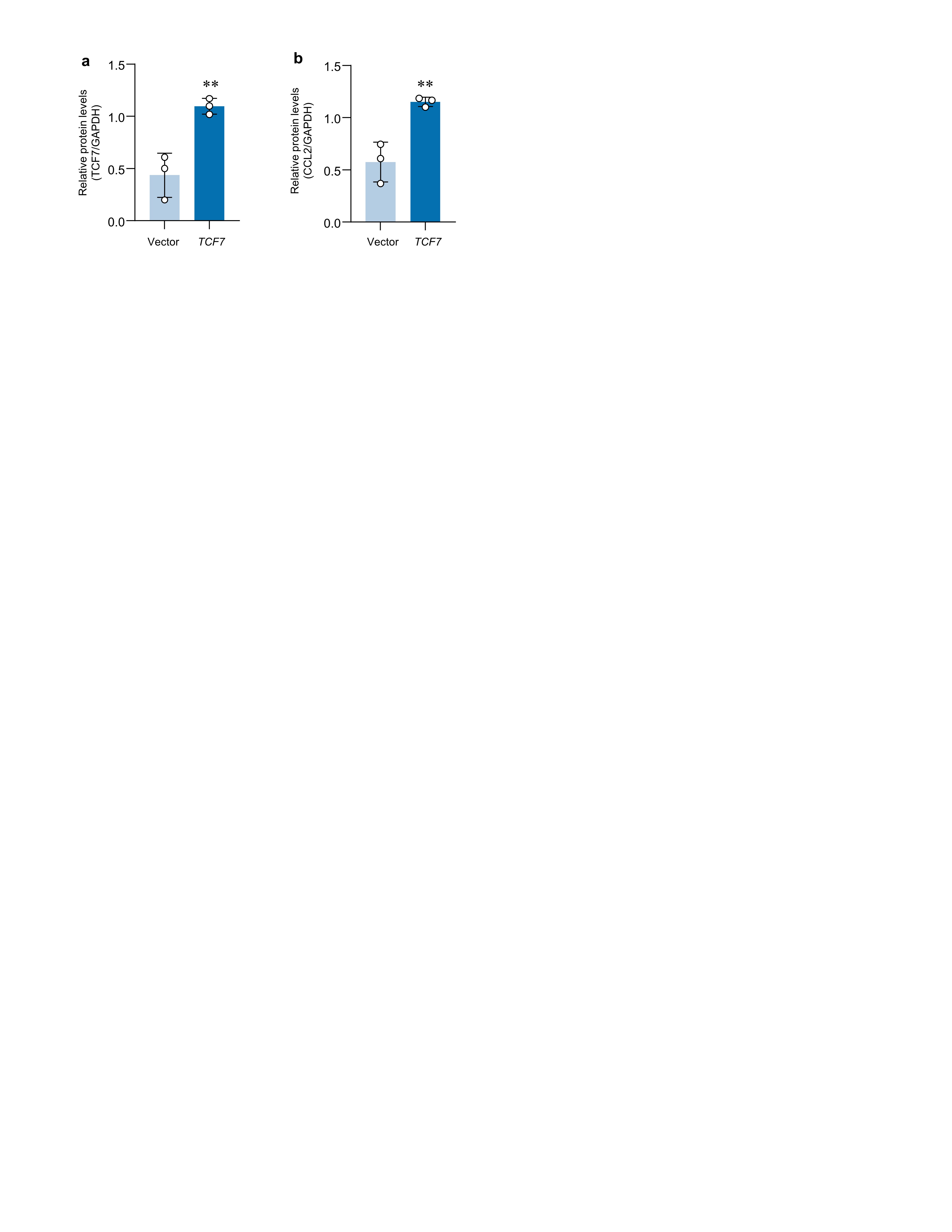
**

**Fig. S8** Quantification of TCF7 and CCL2 protein levels. (**a and b**) The quantification of Western blots of TCF7 (**a**) and CCL2 (**b**) protein levels was determined in C28/I2 cells transfected with *TCF7* expression plasmid (n = 3). GAPDH expression was detected as a loading control. *: *P*<0.05, **: *P*<0.01, ***: *P*<0.001. Data are presented as mean ± SD. Statistical analysis was conducted using two-tailed unpaired Student’s *t*-test (a and b).


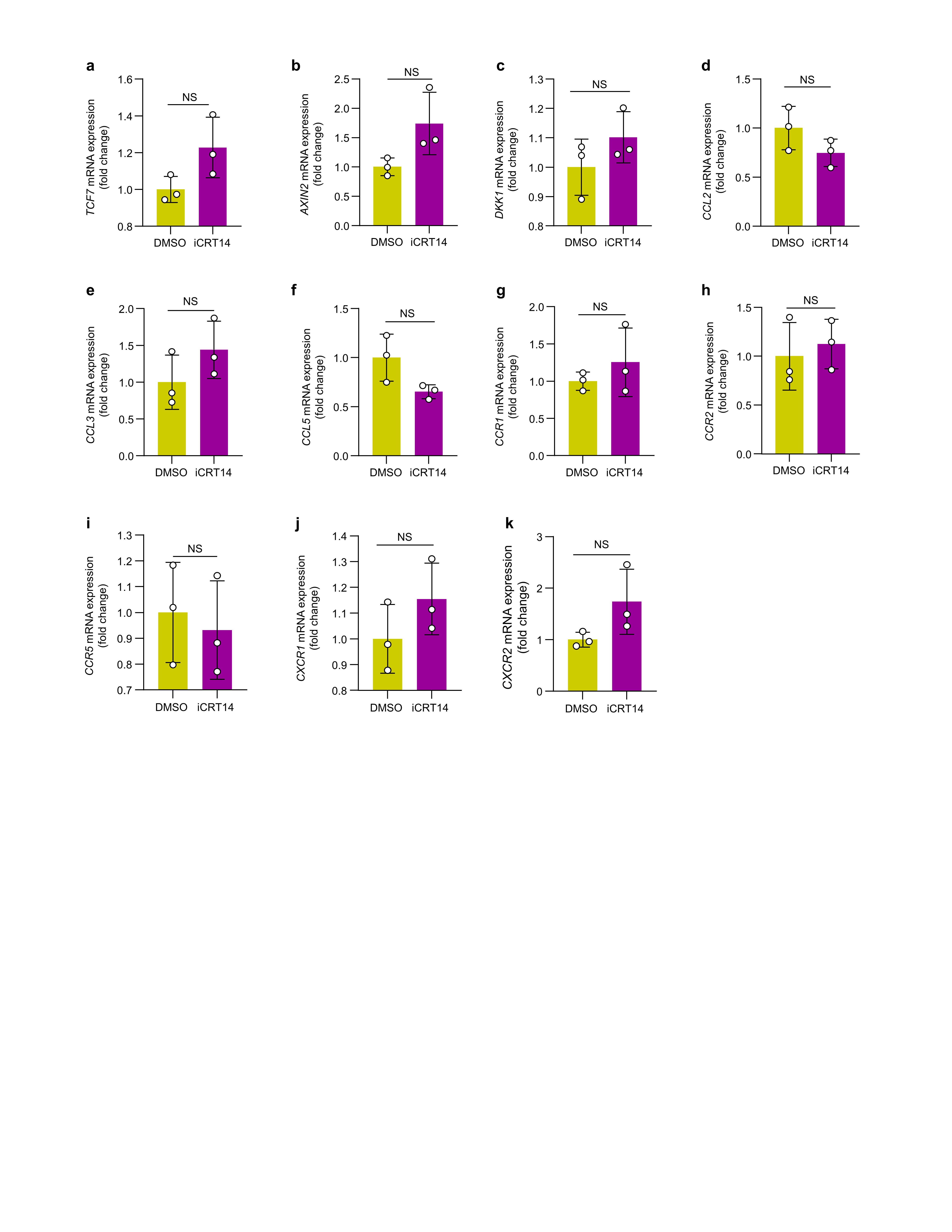


**Fig. S9** β-catenin inhibitor does not affect β-catenin downstream target genes and chemokines expression without IL-1β stimulation. **a**-**k** mRNA expression of *Tcf7* (**a**), *Axin2* (**b**), *Dkk1* (**c**), *Ccl2* (**d**), *Ccl3* (**e**), *Ccl5* (**f**), *Ccr1* (**g**), *Ccr2* (**h**), *Ccr5* (**i**), *Cxcr1* (**j**) and *Cxcr2* (**k**) by qPCR analysis in C28/I2 cells treated with iCRT14 (50 μM), but in the absence of IL-1β stimulation. The mRNA level in DMSO treated control group was arbitrarily set to 1 (n = 3). *: *P*<0.05, **: *P*<0.01, ***: *P*<0.001. Data are presented as mean ± SD. Statistical analysis was conducted by two-tailed unpaired Student’s *t*-test (a-k).


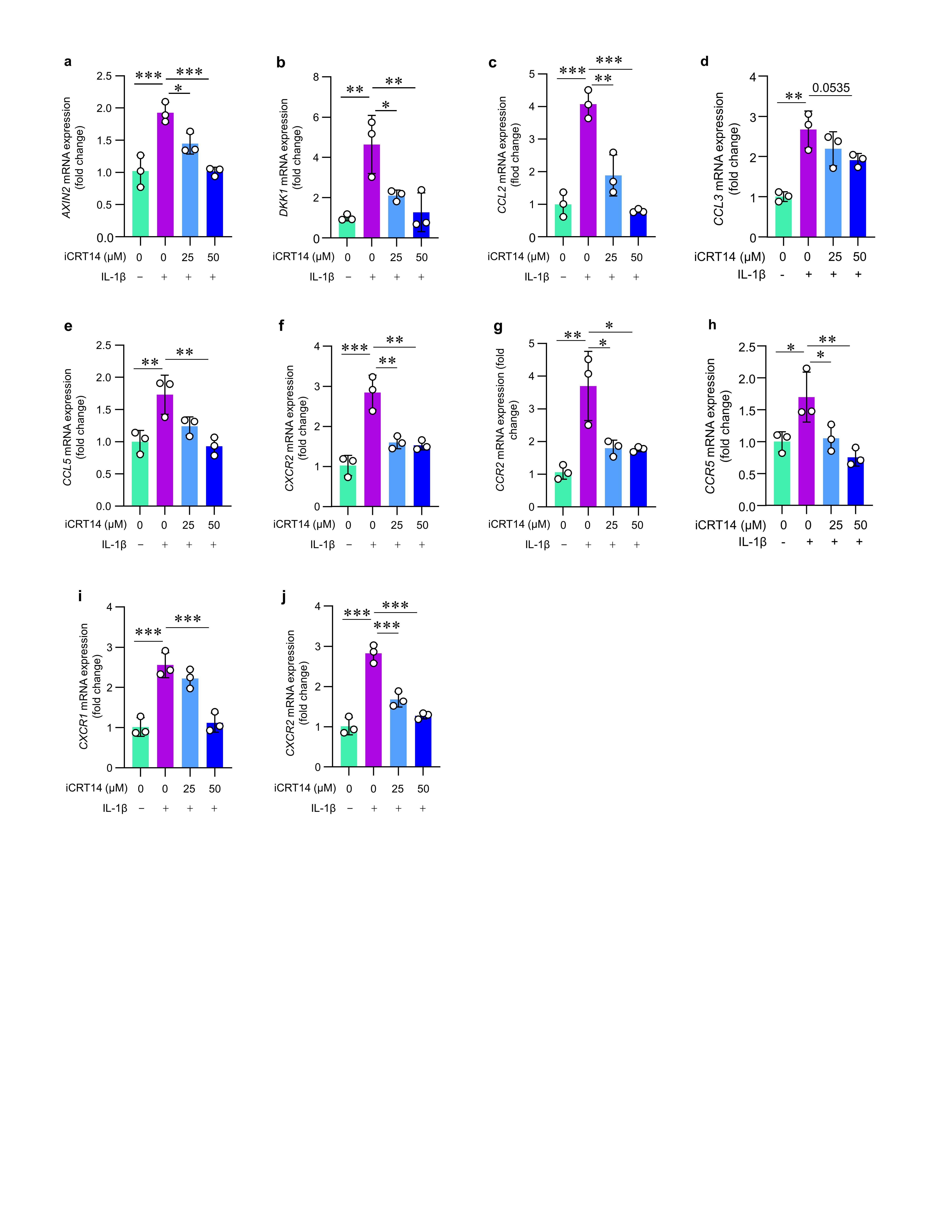


**Fig. S10** Inhibition of β-catenin signaling reverses IL-1β-induced chemokines expression in C28/I2 cells. **a-j** mRNA expression of *Axin2* (**a**), *Dkk1* (**b**), *Ccl2* (**c**), *Ccl3* (**d**), *Ccl5* (**e**), *Ccr1* (**f**), *Ccr2* (**g**), *Ccr5* (**h**), *Cxcr1* (**i**) and *Cxcr2* (**j**) by qPCR analysis in C28/I2 cells treated with IL-1β (20 ng/ml), iCRT14 (0, 25, 50 μM) or both IL-1β and iCRT14, as indicated. The mRNA level in untreated control group was arbitrarily set to 1 (n = 3). *: *P*<0.05, **: *P*<0.01, ***: *P*<0.001. Data are presented as mean ± SD. Statistical analyses were conducted by one-way ANOVA followed by Tukey’s post-test for multiple comparisons (a-j).


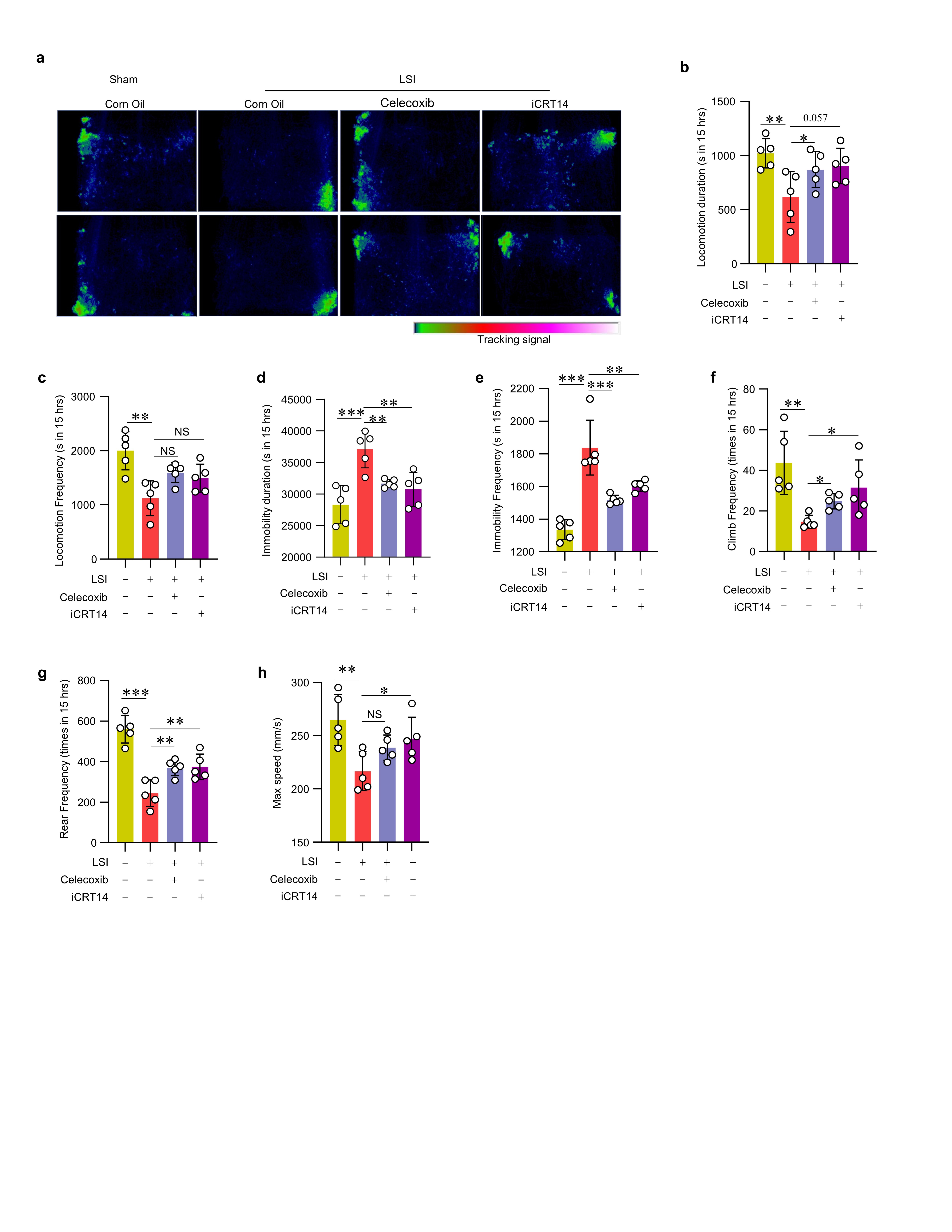


**Fig. S11** Inhibition of β-catenin signaling reverses reduced spontaneous activities caused by LSI surgery. **a** The representative images of mice motion track record of LSI-mice treated with iCRT14 (50 mg/kg) or celecoxib (10 mg/kg) (positive control) (n = 5). Corn oil was used as a negative control. **b-h** The behavior tests using LABORAS showed recovery of spontaneous activities, including locomotion duration (**b**), locomotion frequency (**c**), immobility duration (**d**), immobility frequency (**e**), climb frequency (**f**), rear frequency (**g**) and max speed (**h**), by the treatment with iCRT14 (50 mg/kg) or celecoxib (10 mg/kg) (positive control) (n = 5). *: *P*<0.05, **: *P*<0.01, ***: *P*<0.001. Data are presented as mean ± SD. Statistical analyses was conducted by one-way ANOVA followed by Tukey’s post-test for multiple comparisons (b-h).


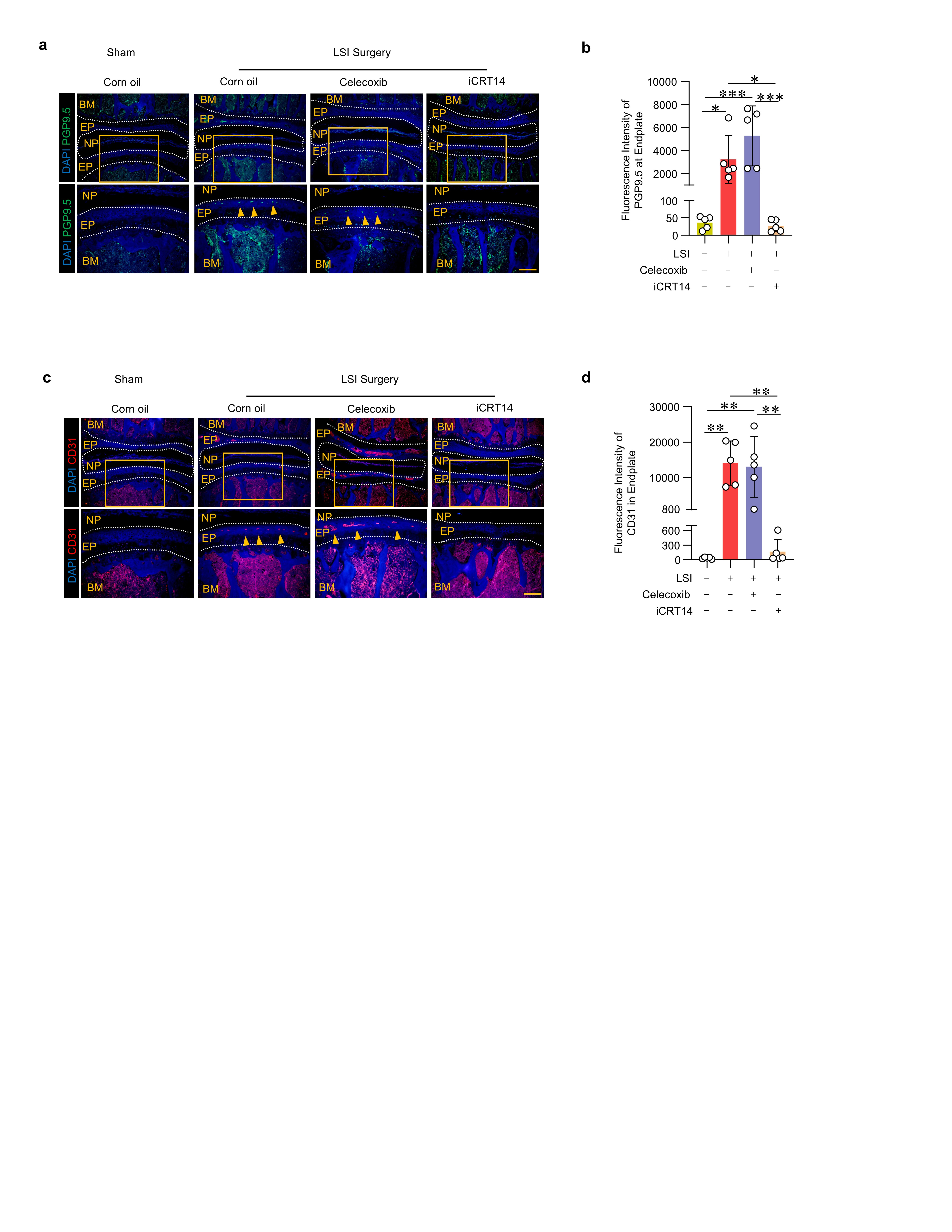


**Fig. S12** Inhibition of β-catenin signaling reverses vascular invasion and nerve ingrowth at the endplate area of the IVD in LSI-mice. **a-d** Representative IF images (scale bar: 50 μm) and quantification of PGP9.5 (**a, b**) and CD31 (**c, d**) expression at the endplate area in LSI-mice treated with iCRT14 (50 mg/kg) or celecoxib (10 mg/kg) (positive control) (n = 5). Arrowheads indicate positive IF signal. BM: bone marrow, EP: endplate, NP: nucleus pulposus. *: *P*<0.05, **: *P*<0.01, ***: *P*<0.001. Data are represented as mean ± SD. Statistical analyses was conducted by one-way ANOVA followed by Tukey’s post-test for multiple comparisons (b and d).

Table S1

**Patient baseline characteristics**

|  | VAS Low group  (VAS score ≤ 5) | VAS High group  (VAS score ≥ 7) |
| --- | --- | --- |
| VAS | 2.889.000±1.745 | 7.00±0.7906*** |
| Gender | 10 male and 8 female | 3 male and 14 female |
| Age | 53±13.56 | 54.93±13.18 |
| BMI | 25.44±4.783 | 24.96±3.240 |
| Modified Pfirrmann Grading System | Grade 3: 8  Grade 4: 4  Grade 6: 1  Grade 7: 2  Grade 5: 4  Grade 5: 5 | Grade 3: 5  Grade 4: 5  Grade 5: 1  Grade 6: 1  Grade 7: 2  Grade 4: 4  Grade 5: 4  Grade 5: 3 |

*** P<0.001

Table S2

**Concentrations and dilutions of antibodies used in this study**

| Antibodies | Method | Concentrations | Dilutions |
| --- | --- | --- | --- |
| PGP9.5 (ab108986) | IF | 0.4 µg/ml | 1:200 |
| Tuj1 (sc-8005) | IF | 1 µg/ml | 1:200 |
| CD31(sc-376764) | IF | 1 µg/ml | 1:200 |
| VEGFA (ab1316) | IF | 5 µg/ml | 1:200 |
| CD34(sc-74499) | IF | 1 µg/ml | 1:200 |
| MMP13(PA5-95486) | IHC | 5 µg/ml | 1:100 |
| Col X(PA5-115039) | IHC | 5 µg/ml | 1:200 |
| Adamts4(NBP1-54369) | IHC | 5 µg/ml | 1:100 |
| Adamts5(ab231595) | IHC | 7.3 µg/ml | 1:100 |
| β-catenin (#9562) | IHC | 1.063 µg/ml | 1:50 |
|  | WB | 0.053 µg/ml | 1:1000 |
| CCL2(NBP1-07035) | IF | 10 µg/ml | 1:100 |
|  | WB | 1 µg/ml | 1:1000 |
| TCF7(sc-271453) | WB | 0.2 µg/ml | 1:1000 |
| GAPDH (D16H11) | WB | 0.034 µg/ml | 1:1000 |

Table S3

**The sequences of primers used in this study**

| Genes | Primer sequence (forward primers) | Primer sequence (reverse primers) |
| --- | --- | --- |
| Ccl2 | CAGCCAGATGCAATCAATGCC | TGGAATCCTGAACCCACTTCT |
| Ccl3 | AGTTCTCTGCATCACTTGCTG | CGGCTTCGCTTGGTTAGGAA |
| Ccl5 | CCAGCAGTCGTCTTTGTCAC | CTCTGGGTTGGCACACACTT |
| Ccr1 | GACTATGACACGACCACAGAGT | CCAACCAGGCCAATGACAAATA |
| Ccr2 | CCACATCTCGTTCTCGGTTTATC | CAGGGAGCACCGTAATCATAATC |
| Ccr5 | TTCTGGGCTCCCTACAACATT | TTGGTCCAACCTGTTAGAGCTA |
| Cxcr1 | CTGACCCAGAAGCGTCACTTG | CCAGGACCTCATAGCAAACTG |
| Cxcr2 | CCTGTCTTACTTTTCCGAAGGAC | TTGCTGTATTGTTGCCCATGT |
| Tcf7 | CTGGCTTCTACTCCCTGACCT | ACCAGAACCTAGCATCAAGGA |
| Dkk1 | CCTTGAACTCGGTTCTCAATTCC | CAATGGTCTGGTACTTATTCCCG |
| Axin2 | TACACTCCTTATTGGGCGATCA | TTGGCTACTCGTAAAGTTTTGGT |
| β-Actin | CATGTACGTTGCTATCCAGGC | CTCCTTAATGTCACGCACGAT |
| Trpa1 | GTCCAGGGCGTTGTCTATCG | AGCACTTCACACGAAGAACCA |
| NGF | TGATCGGCGTACAGGCAGA | GCTGAAGTTTAGTCCAGTGGG |
| Calca | GAGGGCTCTAGCTTGGACAG | AAGGTGTGAAACTTGTTGAGGT |
| Scn9a | CCTTCCTCCGTGACCCTTG | GTAGTCCAATTAGTGCGAACACA |
| TNF-α | CAGGCGGTGCCTATGTCTC | CGATCACCCCGAAGTTCAGTAG |
| IL-1β | GAAATGCCACCTTTTGACAGTG | TGGATGCTCTCATCAGGACAG |
| CCL2 (CHIP1) | GGCCTGAAGGTAAGCTGGCA | GCCCAGACTGACCACAGATGT |
| CCL2 (CHIP2) | GCCATTAACATGCCTCAAGTACTCC | GGAAAGCCTGACTTCATTCCTCAG |
| CCL2 (CHIP3) | GCCTTTGTCCAAGTCTGAAACCC | CCGGCTCCCTGACAATGCAT |
